# Harnessing *Lactiplantibacillus plantarum* EP21 and its membrane vesicles to inhibit myopia development

**DOI:** 10.1080/19490976.2025.2534677

**Published:** 2025-08-01

**Authors:** Chi-Fong Lin, Yu-An Hsu, Yung-Lan Chou, Ying-Chi Chen, En-Shyh Lin, Peng-Tai Tien, Jamie jiin-Yi Chen, Ming-Yen Wu, Chia-Hung Lin, Hui-Ju Lin, Lei Wan

**Affiliations:** a Health Science and Industry, China Medical University, Taichung, Taiwan; b School of Chinese Medicine, China Medical University, Taichung, Taiwan; c Department of Chemistry, National Central University, Taoyuan, Taiwan; d Department of Biomedical Engineering and Environmental Sciences, National Tsing Hua University, Hsinchu, Taiwan; e Department of Veterinary Medicine, National Chung Hsing University, Taichung, Taiwan; f Department of Beauty Science, National Taichung University of Science and Technology, Taichung, Taiwan; g School of Medicine, China Medical University, Taichung, Taiwan; h Eye center, China Medical University Hospital, Taichung, Taiwan; i Department of Medical Laboratory Science and Biotechnology, Asia University, Taichung, Taiwan; j Department of Obstetrics and Gynecology, China Medical University Hospital, Taichung, Taiwan

**Keywords:** *Lactiplantibacillus plantarum* EP21, inflammation, myopia, bacterial membrane vesicle

## Abstract

*Lactiplantibacillus plantarum*, a probiotic that is frequently found in fermented foods, is well known for its numerous health-enhancing properties. This study explored the potential of *Lactiplantibacillus plantarum* EP21 and its membrane vesicles (MVs) in mitigating myopia progression. In animal models of form-deprivation and TGF-β2-induced myopia, EP21 reduced axial elongation and refractive error shifts. EP21 administration suppressed retinal inflammation by inhibiting nuclear factor-κB activation and decreasing expression levels of tumor necrosis factor-α, NLR family pyrin-domain-containing-3, and interleukin (IL)-1β, while upregulating the expression of anti-inflammatory IL-10 in retinal tissues. To identify the molecular mechanism by which EP21 inhibits myopia, purified MVs were administered by intraperitoneal or intravenous injection or applied directly onto the ocular surface. The MVs crossed the blood – retinal barrier and accumulated in the outer segment of the retina. The MVs exhibited anti-myopic properties, indicated by a reduction in axial length elongation and a corresponding upregulation of refractive error. Mechanistic investigations revealed that EP21-MVs contain bacterial miRNAs that inhibit inflammatory responses and retinal structural remodeling. Furthermore, MV and its miRNAs upregulated genes that are associated with ubiquitination (TNF alpha-induced protein 3, TNFAIP3-interacting protein 1, and Tax1 binding protein 1), which are crucial for maintaining ocular proteostasis and regulating inflammation. EP21 administration, besides MV release, altered systemic metabolites, including tryptophan derivatives and short-chain fatty acids. The findings suggest a role of the gut – eye axis in the development of myopia and elucidate a biological pathway whereby gut-derived probiotics and their vesicles can influence ocular health. This research highlights the potential of EP21- and EP21-derived MVs as noninvasive agents for myopia management, and thereby enhances the comprehension of the gut – eye axis. By targeting inflammation and retinal remodeling, probiotics such as EP21 may contribute to addressing the global myopia epidemic, which offers a promising pathway for both preventive and therapeutic strategies.

## Introduction

The global increase in incidence of myopia, especially in East Asia, is high enough to warrant its classification as an epidemic. By 2050, 49.8% (95% CI 43.4%–55.7%) of the global population is expected to become myopic (≤−0.5 D), with 9.8% (95% CI 5.7%–19.4%) experiencing high myopia (≤−5 D).^1^ Owing to the risk of retinal detachment, macular choroidal degeneration, premature cataracts, and glaucoma, high myopia is a crucial risk factor for vision loss. Patients with high myopia (≤−6 D) have a 3.2% annual risk for retinal detachment and the prevalence of macular choroidal neovascularization is nine times higher in these patients.^2,3^ In Taiwan, the incidence of myopia in 6-year-old children is 9.4%, in 15-year-olds is 75%, while that in 18-year-olds is 80%–90%; of these, 10%–20% of these individuals have high myopia (≤−6 D).^3^ Myopia is currently considered a target in the global initiative for the reducing preventable blindness.^4^

The etiopathogenesis of myopia involves both environmental and genetic factors, and inflammation plays a key role. Atropine, with wide clinical usage, slowed myopia progression by reducing the levels of c-Fos, nuclear factor κB (NFκB), interleukin (IL)-6, and tumor necrosis factor (TNF)-α, all of which mediate chronic inflammation.^5^ The impact of inflammatory responses and myopia progression were further substantiated by confirming that allergy-induced inflammation^6^ and air pollutants (particulate matter 2.5)^7^ induced inflammation that exacerbated myopia. Retinal dopamine terminates normal eye growth and plays a pathogenic role in myopia.^8,9^ Dopamine reduced the levels of TNF-α and IL-6 and thereby suppressed inflammation.^10,11^ A pediatric clinical trial of a potent anti-inflammatory agent, crocetin,^12^ demonstrated a beneficial effect in slowing myopia progression.^13^ Despite clinical and experimental data on the association of myopia with inflammation, the origin of the inflammatory responses remains unidentified. No single medical or environmental factor can account for the exceedingly high frequency of myopia, which exceeds 90% among teenagers and young adults in certain regions.^14,15^ Therefore, the identification of the primary cause of myopia and the development of new preventive or therapeutic treatments are indispensable for addressing the myopia epidemic.

Emerging research underscores the significance of the gut microbiota and its metabolites in the pathogenesis and progression of myopia. Studies on animal models, such as mouse and guinea pig, revealed the association of myopia with distinct alterations of the gut microbial composition, which implicated reduced diversity and an imbalance of key bacterial species.^16,17^ High myopia correlated with decreased populations of beneficial microbes such as Akkermansia as well as increased levels of proinflammatory metabolites, such as lipopolysaccharides (LPS),^18,19^ which modulated myopia through systemic inflammatory and metabolic pathways. Microbial metabolites, such as indole-3-acetic acid (3-IAA) – a gut bacteria-produced derivative of tryptophan – plays a protective role by promoting scleral synthesis of type I collagen, to help maintain ocular structural integrity and delay axial elongation, which is associated with high myopia.^18^ Furthermore, microbial imbalance can impair the gut-barrier function and increase systemic inflammation that may accelerate myopic progression through mechanisms such as scleral hypoxia and extracellular matrix remodeling.^16,20^ Metabolomic investigations further elucidated the significance of gut microbiota in the pathogenesis of myopia. Diminished concentrations of anti-inflammatory metabolites, such as gamma-aminobutyric acid (GABA), indicate that alterations of gut metabolic profiles may directly influence inflammatory pathways to modulate myopia progression.^16,21^ Moreover, dopamine-producing gut bacteria and microbiota dysbiosis are linked to myopia. The microbiome in myopia includes bacteria that regulate dopaminergic and GABAergic signaling (e.g., *Clostridium*, *Ruminococcus*, *Bifidobacterium*),^16,18,19^ and these microorganisms may influence neurotransmitter levels and potentially impact eye growth and visual function. This suggests a gut – eye axis that links gut bacteria to myopic progression through dopaminergic pathways.^17,20,22^ However, the mechanisms linking the gut microbiota to myopia remain unclear. Although bacterial metabolites, such as 3-IAA, may affect scleral remodeling, the precise mechanisms that regulate eye structure in myopia,^18^ and whether the bacterial 3-IAA concentrations are sufficient to accumulate adequate myopia-inhibitory levels in the ocular region remain unelucidated.

This study aimed to overcome the limitations in current microbiota – myopia research, which is predominantly cross-sectional and lacks mechanistic insights. The previously suggested link between gut dysbiosis and myopia through inflammatory pathways, causative relationships, and direct therapeutic applications remain unconfirmed. Therefore, this research elucidates the novel use of a probiotic which secretes membrane vesicles (MVs) that can reach ocular tissue and inhibit the pathogenesis of myopia through a targeted, mechanistic approach. By demonstrating the probiotic’s direct effects on eye health, this study could advance the gut – eye axis theory beyond mere correlation to offer a new, biological intervention for myopia management amid the global myopia epidemic.

## Materials and methods

### Animals

Three-week-old male Brown Norway Rats were purchased from the Taiwan National Laboratory Animal Center and National Applied Research Laboratories (NARLabs, Taipei, Taiwan). The rats were raised under a 12-h light/dark cycle. The animal experiment procedures were approved by the Institutional Animal Care and Use Committee of China Medical University (#CMUIACUC-2020–307, #CMUIACUC-2022–196 and #CMUIACUC-2022–196–1) and followed the guidelines for the Use of Animals in Ophthalmic and Vision Research. Monocular form deprivation (MFD) was induced by right eyelid fusion for 3 weeks, and the left eyes served as contralateral control eyes. TGF-β2-induced myopia was induced by TGF-β2 (250 ng/mL) (Cat. # 100–35; Thermo Fisher Scientific, USA) injection (2 μL) on right eyelids (once a week for 3 weeks) while the left eyes served as contralateral control eyes. Fresh cultured live *Lactiplantibacillus plantarum* EP21 were administered by gavage. MVs were administered via intraperitoneal or intravenous injection weekly until the specified total in Figure 3(a–d) was achieved. In the MFD model, rats were divided into control (NC), EP21, MFD or MFD + EP21 groups (*n* = 10 animals each). In the TGF-β2 model, rats were divided into control (NC), EP21, TGF-β2 or TGF-β2 + EP21 groups (*n* = 10 animals each). For MV experiments, rats were divided into control (NC), TGF-β2 or TGF-β2 + MV groups (*n* = 10 animals each). Rats were anesthetized by 2% isoflurane for TGF-β2 and EP21 MVs injection.

### Ocular biometry assessment

Axial lengths were determined by an A-scan ultrasonography (PacScan 300 Plus, New Hyde Park, NY). Refractive error was measured by a retinoscope at the beginning and the end of the experiment. Animals were anesthetized by Zoletil® 50 (5 mg/0.1 kg) (Virbac, France) and dilated the pupil by MYDRIN®-M (SANTEN PHARMACEUTICAL CO, Japan). In each group, ten different measurements were averaged and used in this study. The animals were sacrificed by CO_2_ euthanasia at the end of the study. Retinae were immediately isolated from the eye and used for western blotting analysis. Whole eyeballs were fixed by modified Davidson’s fixative for immunofluorescence staining.

### Bacterial strain isolation

*Lactiplantibacillus plantarum* EP21 was isolated from Golden Syrian hamster feces. Golden Syrian hamster was orally administered a mixture of *Fallopia Japonica* and *Prunella vulgaris*^23^ (100 mg/kg) for 14 days. Feces were dissolved in phosphate-buffered solution (PBS) and subjected to serial dilution before plating onto de Man Rogosa Sharpe (MRS, Cat. # 288130; Becton Dickinson and Company, USA) plates. The isolated bacteria were tested against their anti-myopic effect, whereby *Lactiplantibacillus plantarum* EP21 showed the highest potency. Single colony was amplified and preserved in a glycerol solution with a final concentration of 20% in −80°C. EP21 was continually maintained at a ratio of 1:100 in MRS broth for 18 h, 37°C. EP21 achieved the exponential phase in 6-h incubation and was applied into oral administration.

### Membrane vesicle isolation

After 24-h culture, the supernatant was recovered by centrifuging at 10,000 × g for 1 h. Supernatants underwent a tenfold concentration and were subsequently filtered through a 0.22-μm filter to remove large bacteria debris. MVs were pelleted by ultracentrifugation at 100,000 × g. The MVs were further purified by OptiPrep^TM^ density gradient (Cat. # 1893; Serumwerk, Germany). Density gradient isolation was performed by using a continuous gradient of 45%, 40%, 35%, 30%, 25% and 20% (v/v) OptiPrep in particle-free PBS (Cat. # SH30256.01; Cytiva, USA) at 100,000×g at 4°C for 16 h (SW 90 Ti rotor, Beckman Coulter). The 2-mL fraction was collected from top to bottom (Fractions 1–5). The purified MV pellet was stored in − 80°C.

To determine whether MVs could permeate the blood – retina barrier, purified MVs were stained with 1,1’-Dioctadecyl-3,3,3,’3’-Tetramethylindocarbocyanine Perchlorate (DIL dye; Invitrogen). The DIL-labeled MVs (3.3 × 10^10^ particles) were intravenously injected into C57BL/6 mice. After 24 h, the eyes were harvested and cryosections underwent immunofluorescence microscopy for retinal photography. Live *Lactiplantibacillus plantarum* EP21 were stained with carboxifluorescein diacetate succinimidyl ester (CFSE) and was fed orally for 7 days. The eyes were harvested and cryosections underwent immunofluorescence microscopy for retinal photography.

### Culture of hTERT RPE-1 cell line

RPE-1 cells were acquired from the American Type Culture Collection (CRL-4000 ™, ATCC, USA). Cells were cultured in DMEM:F12 supplemented with 10% fetal bovine serum (FBS, Cat. # 16000044; Thermo Fisher Scientific, USA) and 1% penicillin/streptomycin solution (Cat. # 15140122, Thermo Fisher Scientific, USA) at 37°C in a 5% CO_2_ incubator. To induce short-term NF-κB activation, cells were pretreated with MVs (2×10^6^ particles/well) for 2 h and, then, treated with IL-1β (1.25 ng/mL; Cat. # 200–01, Thermo Fisher Scientific, USA) for 10 min in serum-free DMEM:F12. To induce long-term NF-κB activation, cells were transfected with MV total RNA (MVR; 1.5 μg/mL), miRNA (1 μg/mL), miRNA mixture (0.6 μM for 6 miRNAs probes), Control siRNA (siNC, 0.6 μM, Cat. # sc -37,007) or siRNA mixture (0.6 μM for 3 siRNAs. Cat. # sc -37,655, Cat. # sc -92,019 and Cat. # sc -106,831; Santa Cruz Biotechnology, USA) for 16 h, and then, treated with IL-1β (1.25 ng/mL) or with TNF-α (2.5 ng/mL; Cat. # 300–01, Thermo Fisher Scientific, USA) for 24 h in DMEM:F12 with 10% heat-inactivated FBS. The supernatants were collected to detect IL-6 and IL-8 by enzyme-linked immunosorbent assay.

### Immunofluorescence staining

Eyes were embedded into paraffin or OCT (FSC 22® Clear, Cat. # 3801480; Leica, Germany). Sections were cut at 3-μm thickness in paraffin-embedded blocks while at 15-μm thickness in OCT-embedded samples. Antigens were retrieved from eye sections by Epitope Retrieval Solution (Cat. # RE7113; Leica, Germany) after deparaffinization. Sections were blocked in PBST (137 mM NaCl, 2.7 mM KCl, 10 mM Na_2_HPO_4_, 1.8 mM KH_2_PO_4_, 0.1% Tween-20) buffer containing 1% BSA (Bovine serum albumin, Cat. # A1310–05; USBiological Life Science, USA) and 10% normal rabbit serum and then incubated with the primary antibodies overnight at 4°C. Specific primary antibody includes NF-κB (GeneTex; Cat. # GTX102090; RRID:AB_10630493), TNF-α (Bioworld; Cat. # BS1857; RRID:AB_1662107), NLRP3 (Cell Signaling Technology; Cat. # 13158; RRID:AB_2798134), IL-1β (Abcam; Cat. # ab9722; RRID:AB_308765), IL-10 (Bioss; Cat. # bs-0698 R; RRID:AB_10856552), TGF-β (Abcam; Cat. # ab66043; RRID:AB_1143428), MMP2 (Abcam; Cat. # ab97779; RRID:AB_10696122), COL1A1 (Novus; Cat. # NB600–408; RRID:AB_10000511), ChromPure Rat IgG (Jackson ImmunoResearch Labs; Cat. # 012–000–003; RRID:AB_2337136) and Ubiquitin (Santa Cruz Biotechnology; Cat. # sc-8017; RRID:AB_628423). The slides were incubated with a secondary antibody conjugated to alexa flour 546 for 1 h at room temperature. Nuclei were stained by 4,’6-diamidino-2-phenylindole (DAPI) for 5 min and washed three times with PBS. The images were acquired by using a Leica DMi8 microscope with MetaVue software, version 7.8.8.0 (Molecular Devices, USA). The quantitative results of retina in images were done by using the ImageJ software (NIH images, USA).

### Western blot analysis

Retinae were lysed in RIPA (10 mM Tris-Cl, 100 mM NaCl, 1 mM EDTA, 1 mM EGTA, 1 mM NaF, 20 mM Na_4_P_2_O_7_, 2 mM Na_3_VO_4_, 1% Triton X-100, 10% glycerol, 0.1% sodium dodecyl sulfate, and 0.5% deoxycholate) lysis buffer supplementing with protease inhibitors (Cat. # 784574834; Roche Applied Science, USA) and phosphatase inhibitors (Cat. # 04906837001; Roche Applied Science, USA). The samples were centrifuged to remove undissolved cell debris at 10,000 × g for 30 min (4°C). Protein quantification was performed with Bradford reagent (Cat. # 5000006; Bio Rad, USA). BSA stock (2 mg/mL) (Cat. # 5000206, Bio Rad, USA) was used to measure protein concentration in a range of 1 μg-10 μg. 15 μg protein was separated by 6–15% SDS-PAGE (Sodium dodecyl sulfate-polyacrylamide gel Electrophoresis) based on the molecular weight of the target protein. Separated proteins were transferred to the 0.45 μm PVDF membrane (Cat. # IPVH85R; Millipore, USA) for 50 min. Membranes were blocked with 5% nonfat milk in PBST for 1 h at room temperature, then incubated with the specific primary antibody against phospho-NF-κB (Cell Signaling Technology; Cat. # 3031 (also 3031S, 3031 L); RRID:AB_330559), NF-κB (Cell Signaling Technology; Cat. # 8242 (also 8242P, 8242S); RRID:AB_10859369), TNF-α (GeneTex; Cat. # GTX637058; RRID:AB 3,086,826), NLRP3 (Abcam; Cat. # ab214185; RRID:AB_2819003), IL-1β (Gene Tex; Cat. # GTX636887; RRID:AB 3,086,825), IL-10 (Bioss; Cat. # bs-0698 R; RRID:AB_10856552), TGF-β2 (AbboMax; Cat. # 500–3784; RRID:AB_3086828), MMP2 (Santa Cruz Biotechnology; Cat. # sc -13,595; RRID:AB_627957), COL1A1 (GeneTex; Cat. # GTX26308; RRID:AB_385204), Ubiquitin (Santa Cruz Biotechnology; Cat. # sc-8017; RRID:AB_628423) and GAPDH (GeneTex; Cat. # GTX100118; RRID:AB_1080976). The above antibodies were diluted in 5% BSA in PBST at a ratio of 1:1000. Secondary antibodies (Cat. # AP132P or Cat. # AP124P) diluted in 5% BSA in PBST at a ratio of 1:10000, applying to the membrane according to the species of primary antibodies. Enhanced Chemiluminescence kit (Cat. # WBKLS0500; Millipore, USA) and ImageQuant LAS-4000 Chemiluminescence Imaging System (GE Healthcare, USA) were used to detect and visualize the target protein labeled with horseradish peroxidase (HRP). Immunoblots were analyzed by the ImageJ software (NIH images, USA).

### Enzyme-linked immunosorbent assay

Detection of cytokines was performed by IL-6 (Cat. # 88–7066–22; Thermo Fisher Scientific, USA) and IL-8 (Cat. # 88–8086–22; Thermo Fisher Scientific, USA) ELISA Ready-Set-Go kit following the manufacturer’s instructions. The wavelength was set at 450 nm with the microplate reader SpectraMax® ABS plus (Molecular Devices, USA).

### Transmission electron microscopy (TEM)

*Lactiplantibacillus plantarum* EP21 MVs isolated from OptiPrep^TM^ gradient was resuspended in 1X PBS and was then negatively stained with 1% Phosphotungstic acid (PTA, Cat. # 11827; Alfa Aesar, USA). One drop of *Lactiplantibacillus plantarum* EP21 MVs was coated onto Formvar-carbon Cu 400 mesh grids (Cat. # 01754-F; TED PELLA INC, USA). Excess liquid was absorbed by filter paper and dried the copper mesh in the moisture proof box before analysis. The analysis was performed by JEM 1400 FLASH microscope at the instrument service center of National Chung Hsing University, Taichung, Taiwan. Accelerated voltage was set at 120 kV to observe the morphology of EP21 MVs.

### Scanning electron cryomicroscopy (cryo-SEM)

After 6-h culture, *Lactiplantibacillus plantarum* EP21 were washed three times at 6,000 × g for 15 min, and resuspended in one-tenth the volume of PBS. Next, we added one drop of bacterial suspension onto a piece of a 0.2-μm polycarbonate membrane (Cat. # GTTP02500; Millipore, USA), and freeze the sample into liquid nitrogen immediately. The membrane was coated with platinum and visualized using a JEOL JSM-7800F SEM (Jeol Ltd., Japan) microscope at the instrument service center of National Chung Hsing University, Taichung, Taiwan.

### Nanoparticle tracking analysis (NTA)

Nanoparticle tracking analysis was performed using the Nanoparticle Characterization System, NanoSight (Malvern, UK). Briefly, 200 µL EP21 MVs solution was diluted 4-fold with PBS to obtain a total 1-mL volume. Then, the solution was loaded into the measuring chamber using a syringe. Dynamic videos of nanoparticles were recorded three times for 1 min, and samples were calculated using NanoSight analytical software, version NTA 3.4 (Malvern Instruments, UK). The particle size as well as the concentrations (particles/mL) of EP21 MVs were estimated in triplicate for each sample. We designed the downstream experiment according to this result.

### Quantitative realtime-polymerase chain reaction (qRT-PCR)

Total RNA was extracted using a RNeasy mini Kit (Cat. # 74106; Qiagen, Germany) according to the manufacturer’s instructions. Purity of RNA was determined in terms of the A260/A280 ratio by a Nanodrop spectrophotometer (NanoVue Plus, Biochrom, USA). Complementary DNA was synthesized using a Reverse Transcriptase Kit (Cat. # 1708890, BIO-RAD, USA). The primer sequences for each gene were listed in Supplementary Table S1. The transcript levels of genes were quantified with RT-qPCR using the cDNA as a template in a StepOne Plus system (Applied Biosystems) with SsoAdvanced Universal SYBR Green Supermix (Cat. # 1725272; BIO-RAD, USA). The relative expression of each mRNA was calculated according to 2^−ΔΔCt^ with glyceraldehyde-3-phosphate dehydrogenase (GAPDH) as the internal control.

### Quantitation and statistical analysis

GraphPad Prism (Version 9.0) was used to analyze all the data. Intergroup differences were analyzed by using the unpaired *t*-test and one-way ANOVA. *p* < 0.05 was set as the threshold for statistical significance.

## Results

### *Inhibition of myopia pathogenesis by* Lactiplantibacillus Plantarum *EP21 administration*

**Figure 1.**
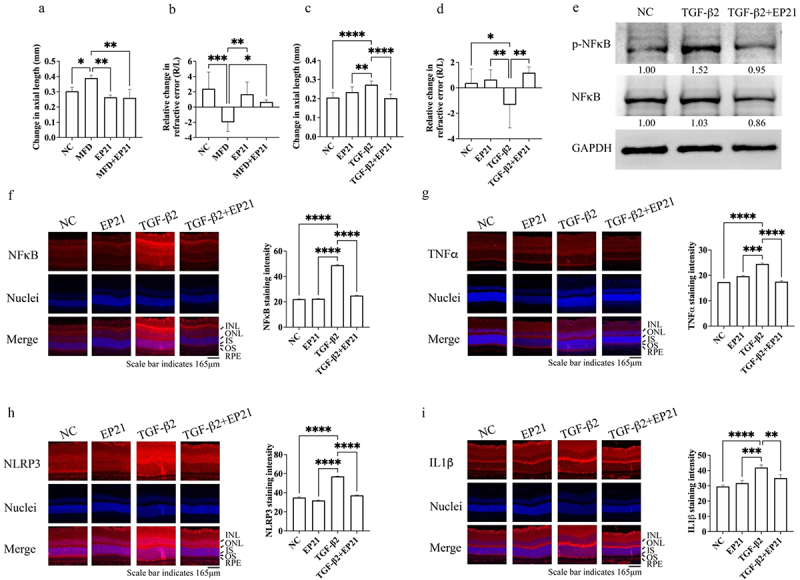
Probiotic treatment suppresses the development of myopia by lowering the inflammatory responses and inflammasome activation. (a) Administration of *Lactiplantibacillus plantarum* EP21 (EP21) mitigated myopia induced by monocular form deprivation (MFD). EP21 mitigated the elongation of axial length provoked by MFD. (b) EP21 prevented the down-regulation of refractive error caused by MFD. The relative change in refractive error of the right eye was standardized to the refractive error of the left eye, which remained untreated. (c) EP21 prevented the elongation of axial length induced by the sub-junctival injection of transforming growth factor-β2 (TGF-β2). (d) EP21 inhibited the down-regulation of refractive error induced by TGF-β2. The relative change in refractive error of the right eye was standardized to the refractive error of the left eye, which remained untreated. (e) NFκB expression levels determined using western blotting. Relative expression levels of p-NFκB and NF-κB (below the lanes) were normalized to glyceraldehyde-3-phosphate dehydrogenase (GAPDH) and the expression levels in lane NC are designated as 1. Immunofluorescence staining of NFκB (f), tumor necrosis factor α (TNFα) (g), nucleotide-binding oligomerization domain leucine-rich repeat and pyrin domain-containing protein 3 (NLRP3) (h), and interleukin 1β (IL1β) (i) expression in the eyes of NC, EP21, TGF-β2 and TGF-β2 + EP21-treated rats. Results are shown as mean ± SD. The scale bar indicates 165 μm and 200× magnification for if staining images. Relative expression levels were determined using image J software. Analysis of variance was applied to examine for significant differences (*p* < 0.05), and Tukey’s multiple comparison tests were used for pairwise comparisons. NC: negative control. INL: inner nuclear layer; ONL: outer nuclear layer; IS: inner segment; OS: outer segment; RPE: retinal pigment epithelium.

*Lactiplantibacillus Plantarum* EP21 (EP21), a probiotic isolated from Syrian hamsters that were fed a herbal formulation to treat monocular form deprivation (MFD)-induced myopia, was orally administered daily to Brown Norway rats for 21 days. The axial length and refractive error were measured both before and after the treatment. The change in axial length and refractive error of MFD was compared to the values of the negative control (NC), EP21, and MFD + EP21 groups to evaluate the treatment efficacy. The rats in the MFD group developed myopia in their right eye (change in axial length: 0.39 ± 0.02 mm), whereas the rats in the NC, EP21, and MFD + EP21 arms did not (change in axial length: 0.30 ± 0.03, 0.26 ± 0.02, and 0.24 ± 0.05 mm, respectively; Figure 1a). Moreover, the refractive error of MFD eyes was significantly lower than that of NC, EP21, and MFD + EP21 eyes (change in refractive error: −2.00 ± 0.02, 2.4 ± 2.19, 1.68 ± 1.60, and 0.67 ± 0.28 D for MFD, NC, EP21, and MFD + EP21, respectively; Figure 1b). The efficacy of EP21 in inhibiting the pathogenesis of myopia was further evaluated in a TGF-β2-induced myopia model,^24,25^ wherein EP21 was orally administered daily to EP21 only (rats without TGF-β2 administration) and TGF-β2 + EP21 rats for 21 days. To evaluate treatment efficacy, the change in axial length and refractive error of TGF-β2 was compared to the values of the NC, EP21, and TGF-β2 + EP21 groups. The rats in the TGF-β2 group developed myopia in their right eye (change in axial length: 0.27 ± 0.02 mm), whereas the rats in the NC, EP21, and TGF-β2 + EP21 did not (change in axial length: 0.21 ± 0.03, 0.23 ± 0.03, and 0.20 ± 0.02 mm, respectively; Figure 1c). Furthermore, the refractive error of TGF-β2 eyes was significantly lower than that of the other eyes (change in refractive error: −1.33 ± 1.81, 0.38 ± 1.10, 0.65 ± 0.77, and 1.19 ± 0.46 D for TGF-β2, NC, EP21, and TGF-β2 + EP21, respectively; Figure 1d). Myopic eyes had higher expression of TGF-β (Supplementary Figure S1A) and metalloproteinase 2 (MMP2; Supplementary Figure S1B), and downregulated collagen I expression (COL1A1; Supplementary Figure S1C), which are consistent with previous research.^5^ EP21 administration reduced TGF-β and MMP2 expression and increased COL1A1 expression. Next, we assessed the retinal anti-inflammatory properties of EP21. EP21 administration significantly inhibited NFκB activation and expression, which is otherwise induced by TGF-β2 in the retina (Figure 1e,f). Furthermore, EP21 administration mitigated the TGF-β2-stimulated retinal expression of the proinflammatory cytokine TNFα (Figure 1g). The activation of the TGF-β2-stimulated inflammasome was attenuated by EP21, as evidenced by the suppression of NOD-, LRR-, and pyrin domain-containing protein 3 (NLRP3; Figure 1h) and IL1β (Figure 1i) expression in the retina. Moreover, compared to the group treated solely with TGF-β2, EP21 induced upregulation of the anti-inflammatory cytokine IL-10 (Supplementary Figure S1D).

### L. Plantarum *EP21-MVs reduced inflammation and crossed the blood – retinal – barrier (BRB)*

**Figure 2.**
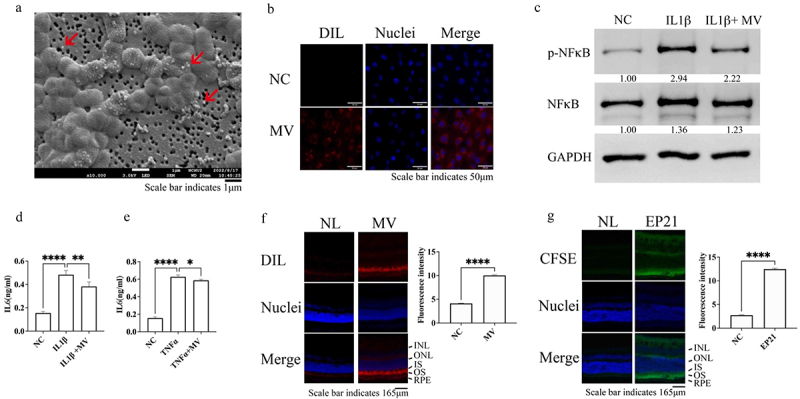
Membrane vesicles secreted by *Lactiplantibacillus plantarum* EP21 (MV) inhibit inflammatory responses and pass across blood-retinal-barrier. (a) *Lactiplantibacillus plantarum* EP21 and its derived MVs (red arrows) detected by cryo-scanning electron microscopy. (b) DIL-labelled MVs were internalized by retinal pigment epithelial (RPE-1) cells. (c) EP21 MVs inhibited interleukin (IL) 1β induced nuclear factor (NF)κB activation in RPE-1 cells. NFκB expression levels determined using western blotting. Relative expression levels of p-NF-κB and NF-κB (below the lanes) were normalized to glyceraldehyde-3-phosphate dehydrogenase (GAPDH) and the expression levels in lane NC are designated as 1. (d) EP21 MVs inhibited IL1β induced IL6 expression in RPE-1 cells. Cells were treated with phosphate buffered saline (NC), IL1β or IL1β + MV for 24 hours. The IL6 concentration was then determined by enzyme-linked immunosorbent assay. (e) EP21 MVs inhibited tumor necrosis factor α (TNFα) induced IL6 expression in RPE-1 cells. Cells were treated with NC, TNFα or TNFα + MV for 24 hours. The IL6 concentration was then determined by enzyme-linked immunosorbent assay. For (d) and (e), analysis of variance was applied to examine for significant differences (*p* < 0.05), and Tukey’s multiple comparison tests were used for pairwise comparisons. (f) Dil-labeled or non-labeled EP21 MVs were inject intravenously into C57BL/6 mice. After 24 hours, eyes were collected to determine the fluorescence intensities in the retina. (g) EP21 was stained with or without carboxifluorescein diacetate succinimidyl ester (CFSE) and orally fed C57BL/6 mice for 7 days. Eyes were collected to determine the fluorescence intensities in the retina. For (f) and (g), relative expression levels were determined using image J software. T-test was used to evaluate the significant difference between non-labeled (NL) and EP21.

To elucidate the mechanism whereby EP21 inhibits myopia progression, we isolated EP21-derived MVs (Figure 2a) and separated these into five distinct fractions for the transmission electron microscopy (TEM)-based assessment of their structural integrity. As demonstrated in Supplementary Figure S2A, because fraction 5 predominantly comprised compromised MVs, fractions 1–4 were pooled for subsequent experimental procedures. The mean diameter of EP21-MVs was measured at 152.7 ± 1.0 nm (Supplementary Figure S2B) and EP21-MV internalization by retinal pigment epithelial cells (RPE-1 cells) was determined (Figure 2b) and shown to inhibit IL-1β-induced NFκB expression and activation (Figure 2c). Besides RPE-1 cells, IL-1β- and MV-treated ARPE-19 (Supplementary Figure S3A) and two primary RPE cells provided by two different vendors (Supplementary Figure S3B and C) showed the same results. IL-1β and TNFα-stimulated expression of IL-6 (Figures 2d,e, respectively) and IL-8 (Supplementary Figures S4A and B, respectively) was suppressed following EP21-MV treatment. To investigate whether EP21-MVs can traverse the BRB and permeate the retina, DIL-labeled MVs or unlabeled MVs were intravenously injected into C57BL/6 mice. Compared to the unlabeled MV-injected retina, the DIL-labeled MV-injected retina exhibited a significant accumulation of fluorescence within the outer segment (OS; Figure 2f). It was noteworthy to ascertain whether analogous translocation phenomena were observable following the ingestion of live EP21. EP21 was stained with carboxifluorescein diacetate succinimidyl ester (CFSE) and administered daily via oral gavage to C57BL/6 mice for 7 consecutive days. Our investigation revealed a statistically significant elevation in fluorescence within the retina of mice that were administered CFSE-labeled EP21 as compared to those that received unlabeled EP21 (Figure 2g). Furthermore, an enhanced fluorescence signal was observed in both hepatic (Supplementary Figure S5A) and cerebral (Supplementary Figure S5B) tissues in mice that received CFSE-labeled EP21 administration compared to those that received unlabeled EP21.

### L. Plantarum *EP21-MV administration inhibited myopia development*

**Figure 3.**
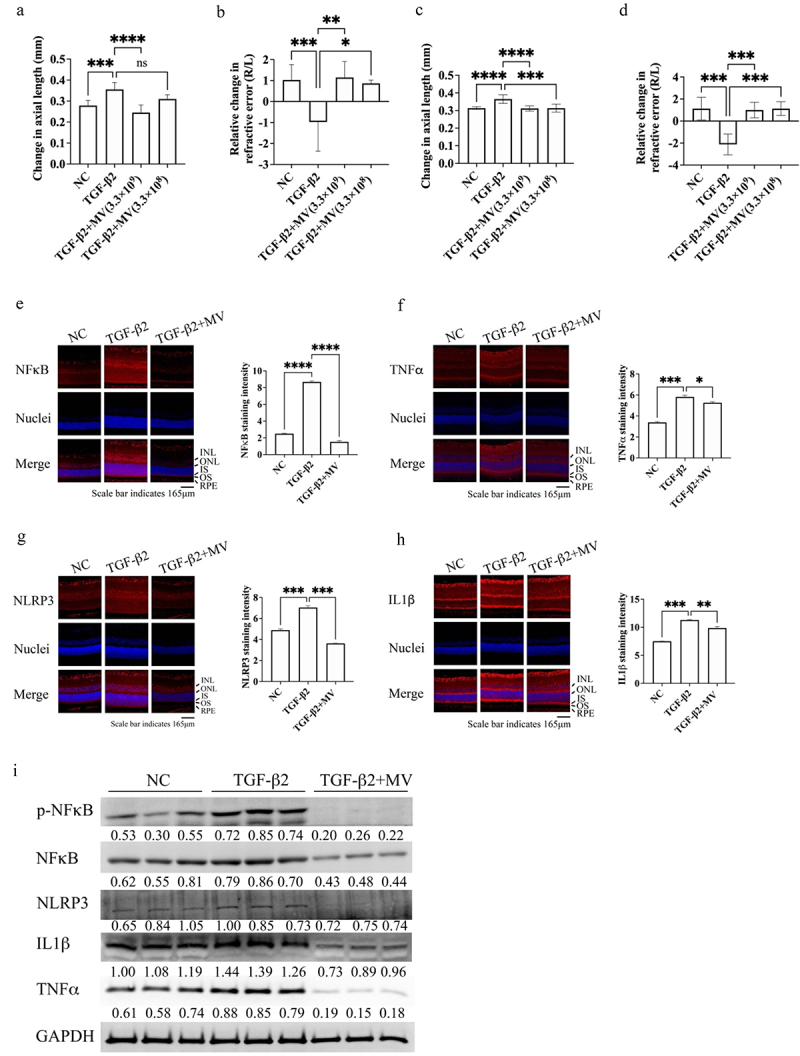
*Lactiplantibacillus plantarum* EP21 membrane vesicles (EP21 MVs) treatment suppresses the development of myopia by lowering the inflammatory responses and inflammasome activation. Myopia was induced by sub-junctival injection of transforming growth factor-β2 (TGF-β2). (a) Intraperitoneal injection of EP21 MVs (3.3 × 10^8^ or 3.3 × 10^9^ particles) prevented the elongation of axial length caused by the sub-junctival injection of TGF-β2. (b) Intraperitoneal injection of EP21 MVs inhibited the down-regulation of refractive error induced by TGF-β2. The relative change in refractive error of the right eye was standardized to the refractive error of the left eye, which remained untreated. (c) Intravenously injection of EP21 MVs (3.3 × 10^8^ or 3.3 × 10^9^ particles) prevented the elongation of axial length caused by the sub-junctival injection of TGF-β2. (d) Intravenously injection of EP21 MVs inhibited the down-regulation of refractive error induced by TGF-β2. The relative change in refractive error of the right eye was standardized to the refractive error of the left eye, which remained untreated. Immunofluorescence staining of NFκB (e), tumor necrosis factor α (TNFα) (f), nucleotide-binding oligomerization domain leucine-rich repeat and pyrin domain-containing protein 3 (NLRP3) (g), and interleukin 1β (IL1β) (h) expression in the eyes of negative control (NC), TGF-β2 and TGF-β2 + EP21 MV-treated (intraperitoneally administered) rats. Results are shown as mean ± SD. The scale bar indicates 165 μm and 200× magnification for if staining images. Relative expression levels were determined using image J software. Analysis of variance was applied to examine for significant differences (*p* < 0.05), and Tukey’s multiple comparison tests were used for pairwise comparisons. INL: inner nuclear layer; ONL: outer nuclear layer; IS: inner segment; OS: outer segment; RPE: retinal pigment epithelium. (h) The expression levels of phospho-NFκB (p-NFκB), NFκB, NLRP3, pro-IL1β, and TNFα in the retina were determined using western blotting. Relative expression levels (below the lanes) were normalized to glyceraldehyde-3-phosphate dehydrogenase (GAPDH).

Purified MVs from EP21 were administered via intraperitoneal injection (IP) into Brown Norway rats, which had TGF-β2-induced myopia. To assess treatment efficacy, the TGF-β2-associated alterations in axial length and refractive error with were compared to the measurements obtained from the negative control (NC) and the TGF-β2 + MV groups. The rats in the TGF-β2 group exhibited longer axial length in their right eye, in contrast to rats in the NC and TGF-β2 + MV groups (Figure 3a). Furthermore, the refractive error of the TGF-β2-treated eyes was significantly lower compared to that of the NC and TGF-β2 + MV-treated eyes (Figure 3b). Consistent findings were observed with the intravenous (IV) administration of MV, as evidenced by the reduced axial length (Figure 3c) and elevated refractive error (Figure 3d) in eyes subjected to MV treatment compared to eyes treated solely with TGF-β2. Moreover, the anti-myopic effect of MV was confirmed by applying MV directly as eyedrops, which induced shorter axial length and higher refractive error (Supplementary Figure S6A and B, respectively). IP administration of MV significantly reduced retinal TGF-β2-induced NFκB expression and activation (Figure 3e,i). Furthermore, MV administration decreased retinal TGF-β2-stimulated TNFα expression (Figure 3f,i) and mitigated TGF-β2-induced inflammasome activation, as indicated by reduced retinal NLRP3 (Figure 3g,i) and IL-1β (Figure 3h,i) expression. Furthermore, IL-10 levels increased with MV treatment compared to the TGF-β2-only group (Supplementary Figure S7A and B). MV administration inhibited the levels of TGF-β (Supplementary Figure S7C and F) and MMP2 (Supplementary Figure S7D and F) and promoted COL1A1 (Supplementary Figure S7E and F) expression.

### *RNAs in* L. Plantarum *EP21 membrane vesicles (MVR) modulate inflammatory response*

**Figure 4.**
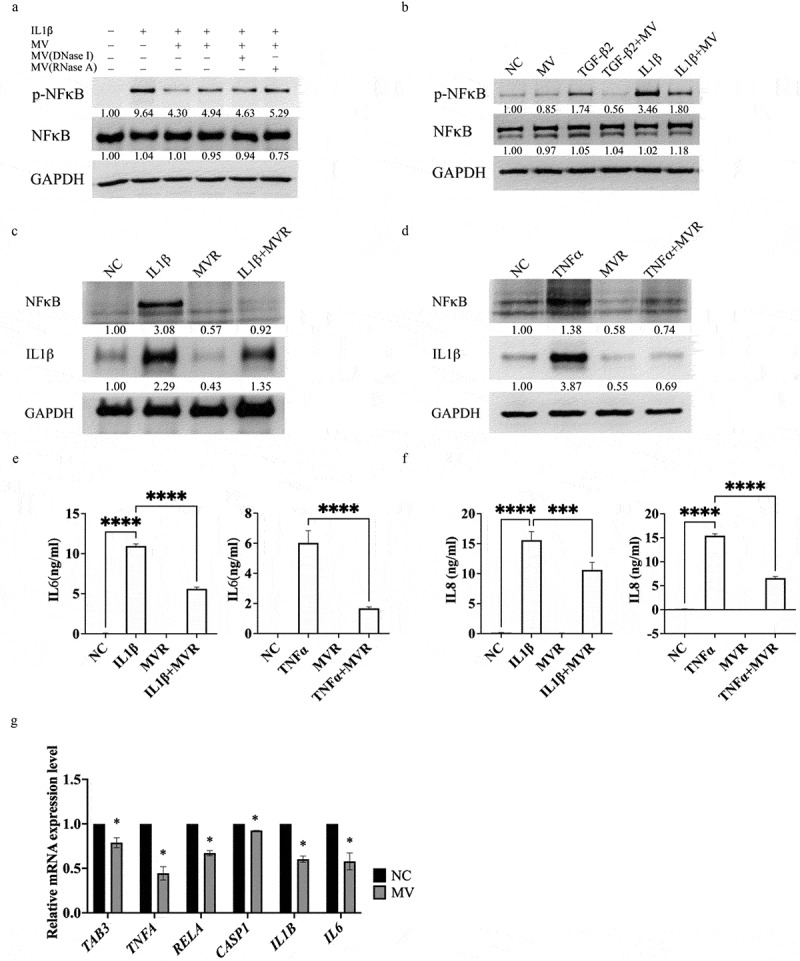
RNA in the *Lactiplantibacillus plantarum* EP21 membrane vesicles (EP21 MVs) inhibited inflammatory reactions in the retinal pigment epithelial cell. (a) Mediators inside the EP21 MVs but not DNA or RNA associated with MVs inhibited the activation of nuclear factor κB (NFκB). Intact EP21 MVs were treated with DNase I or RNase a to remove DNA or RNA binds to the surface of EP21 MVs. RPE-1 cells were treated with phosphate buffered saline (NC), interleukin 1β (IL1β), MV, DNAase I or RNase a treated MVs for 30 min and the activation of NFκB was determined by western blot. Relative expression levels (below the lanes) were normalized to glyceraldehyde-3-phosphate dehydrogenase (GAPDH). (b) The activation of NFκB by transforming growth factor β2 (TGF-β2) or IL1β in RPE-1 cells was inhibited by EP21 MV (MV). RPE-1 cells were treated with indicated treatments for 30 min and the activation of NFκB was determined by western blot. Relative expression levels (below the lanes) were normalized to GAPDH. (c) The increased in expression levels of NFκB and IL1β induced by IL1β in RPE-1 cells were inhibited by transfecting EP21 MV total RNA (MVR). RPE-1 cells were treated with indicated treatments for 24 hours and the expression of NFκB was determined by western blot. Relative expression levels (below the lanes) were normalized to GAPDH. (d) The increased in expression levels of NFκB and IL1β induced by tumor necrosis factor α (TNFα) in RPE-1 cells were inhibited by transfecting EP21 MV total RNA (MVR). RPE-1 cells were treated with indicated treatments for 24 hours and the expression of NFκB was determined by western blot. Relative expression levels (below the lanes) were normalized to GAPDH. (e) MVR inhibited IL1β induced IL6 expression in RPE-1 cells. Cells were treated with phosphate buffered saline (NC), IL1β, MVR, or IL1β + MVR for 24 hours. The IL6 concentration was then determined by enzyme-linked immunosorbent assay. (f) MVR inhibited TNFα induced IL6 expression in RPE-1 cells. Cells were treated with phosphate buffered saline (NC), TNFα, MVR, or TNFα + MVR for 24 hours. The IL6 concentration was then determined by enzyme-linked immunosorbent assay. (g) EP21 MVs inhibited the expression of inflammatory related markers in RPE-1 cells determined by realtime-quantitative polymerase chain reaction. TGF-Beta activated kinase 1 (MAP3K7) binding protein 3:TAB3; tumor necrosis factor; TNFA; RELA proto-oncogene, NF-KB subunit: RELA; caspase 1: CASP1; interleukin 1β: IL1B; interleukin 6:IL6.

MVs contain a multitude of bacterial components that may exhibit immunomodulatory functions, which involves bacterial DNA and RNA. To elucidate the MV-functional components that suppress inflammatory responses and myopia progression, we subjected the MV to treatment with DNase and RNase, to eliminate the DNA and RNA located on the external membranous layer of the MV membrane. However, this did not adversely affect the IL-1β-induced inhibition of NFκB activation (Figure 4a) in RPE-1 cells or TNFα expression in a human microglial cell line (HMC-3; Supplementary Figure S8). MV inhibited TGF-β2-induced NFκB activation in RPE-1 cells (Figure 4b). These findings indicated that the anti-inflammatory and anti-myopic properties of MVs did not stem from DNA or RNA associated with the MV external membranous layer. Subsequently, we isolated the total DNA and RNA from the MVs; DNA could not be detected within the MV, whereas a substantial quantity of RNA was present (Supplementary Figure S9). MV RNA (MVR) demonstrated an inhibitory effect on IL1β- (Figure 4c) and TNFα- (Figure 4d) induced expression of NFκB and IL1β subsequent to its transfection into RPE-1 cells. Moreover, the transfection of MVR significantly suppressed the expression of IL6 (Figure 4e) and IL8 (Figure 4f) that was induced by IL1β and TNFα in RPE-1 cells.

To determine the RNA sequence that possibly modulated gene expression, MVs were transfected into two different primary RPE cells and were subjected to total RNA sequencing (RNAseq). The identified miRNA sequences were blasted against the whole *L. plantarum* genome in the National Library for Biotechnology Information microbial genome database; results showed that there are six miRNAs that may potentially originate from *L. plantarum* (Table 1). A RNase protection assay was performed and confirmed that the miRNAs were presented in EP21 and MV total RNAs but not in methicillin-resistant *Staphylococcus aureus* total RNA (Supplementary Figure S10; Supplementary Table S2). The RNAseq experiments identified several inflammatory genes were inhibited by MV treatment including TGF-Beta Activated Kinase 1 (MAP3K7) Binding Protein 3 (TAB3); tumor necrosis factor α (TNFA); RELA Proto-Oncogene, NFκB Subunit (RELA); caspase 1 (CASP1); interleukin 1β (IL1B) and interleukin 6 (IL6) (Supplementary Figure S11). The inhibitory effect of MV on those inflammatory related genes was confirmed in RPE-1 cells (Figure 4g).

**Table 1.** miRnas found membrane vesicles (MVs) of *Lactiplantibacillus plantarum* EP21.

miRNA	Sequence
NovelmiRNA-284	UAGGUCACUGGGGUCAGAGCCA
NovelmiRNA-487	UCCCCAGUACCCCCACCA
NovelmiRNA-491	CCCUGUCCUCCAGGAGCUCAC
NovelmiRNA-213	GUAGGUGGCCUGACUGGCA
NovelmiRNA-139	CCCAUACUUUCACCCCUCUCU
NovelmiRNA-85	GUCCCUGUUCAGGCGCCA

**Figure 5.**
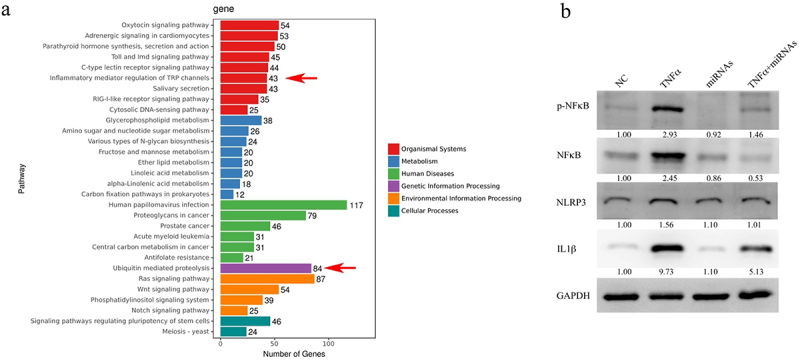
Lactiplantibacillus plantarum EP21 membrane vesicles (EP21 MV) modify ubiquitination pathways. (a) Primary retinal pigment epithelial cells were treated with EP21 MVs for 2 hours. The total RNA of treated cells was subjected to whole transcriptome sequencing analysis. Pathway analysis on the six EP21 miRNAs regulated genes. EP21 miRNAs modulated inflammation and ubiquitination pathways (red arrows). (b) RPE-1 cells were transfected with the mixture of six EP21 miRNAs for 16 hours and the expression level of phospho-nuclear factor κB (p-NFκB), NFκB, nucleotide-binding oligomerization domain leucine-rich repeat and pyrin domain-containing protein 3 (NLRP3) and interleukin 1β (IL1β) was determined by western blot. Relative expression levels were determined using image J software. Relative expression levels (below the lanes) were normalized to glyceraldehyde-3-phosphate dehydrogenase (GAPDH).

The KEGG pathway analysis elucidated that the six identified miRNAs modulate multiple inflammation-associated pathways (Figure 5a). A mixture of the six miRNAs was introduced into RPE-1 cells to assess the anti-inflammatory effects induced by TNFα treatment. As depicted in Figure 5b, the mixture of miRNAs significantly attenuated both the activation and expression of NFκB, as well as the expression of IL-1β, compared to cells treated with transfection reagents without miRNAs. Furthermore, we assessed the individual activities of each miRNA, and the findings revealed that, with the exception of miRNA-491, all other miRNAs inhibited the expression and activation of NFκB when compared to cells treated with transfection reagents without miRNAs (Supplementary Figure S12A). miRNA-284, 213, 139, and 85 demonstrated a significant reduction in the expression of NLRP3 and IL-1β relative to cells treated with transfection reagents without miRNAs (Supplementary Figure S12A). All investigated miRNAs decreased IL6 (Supplementary Figure S12B) and IL8 (Supplementary Figure S12C) expression levels in RPE-1 cells treated with TNFα. The results suggested that the biological efficacy of MV derives from the combinatorial effect of the miRNAs rather than individual miRNAs.

### *Altered protein ubiquitination by* L. plantarum *EP21-MVs*

**Figure 6.**
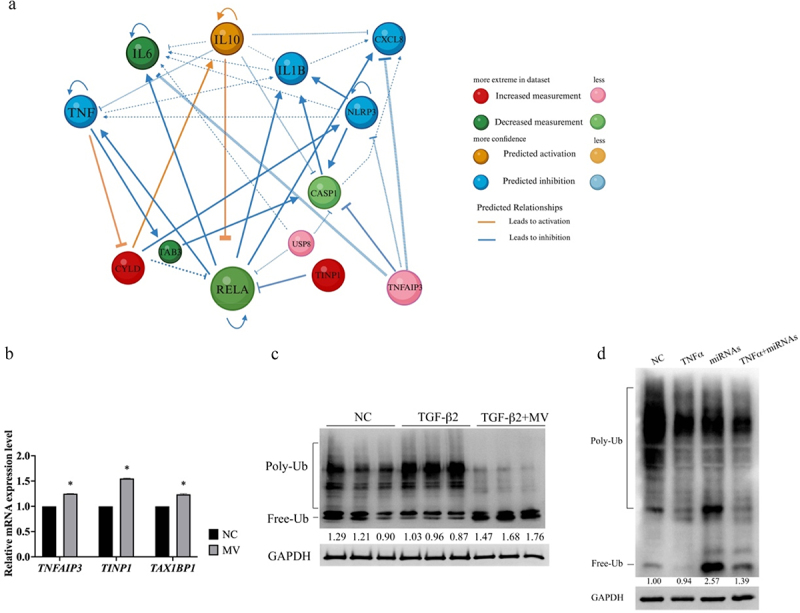
*Lactiplantibacillus plantarum* EP21 membrane vesicles containing miRNAs modify inflammation and ubiquitination pathways. (a) Ingenuity pathway analysis identified the gene networks associated with inflammation and ubiquitination pathways. (b) The levels of genes associated with ubiquitination, specifically TNF alpha induced protein 3 (TNFAIP3), TNFAIP3-interacting protein 1 (TINP1), and Tax1 binding protein 1 (TAX1BP1), were significantly elevated in RPE-1 cells following treatment with EP21 MV. NC refers to the negative control condition. (c) Total protein was extracted from the retina of eyes subjected to negative control, transforming growth factor β2 (TGF-β2), and EP21 MVs treatment, and the extent of protein ubiquitination was assessed using western blot analysis. (d) RPE-1 cells underwent treatment with tumor necrosis factor (TNF) α, transfection with miRNAs or co-treatment with TNFα and miRNAs for a duration of 24 hours. The level of protein ubiquitination was evaluated through western blotting. Poly-ub: polyubiquitinated protein; free-ub: free ubiquitin. Relative free ubiquitin expression levels were determined using image J software. Relative expression levels (below the lanes) were normalized to glyceraldehyde-3-phosphate dehydrogenase (GAPDH).

The RNAseq results were further analyzed by utilizing the Ingenuity Pathway Analysis, which revealed the upregulation of genes associated with protein ubiquitination including TNF alpha induced protein 3 (*TNFAIP3*), TNFAIP3-interacting protein 1 (*TINP1*), and Tax1 binding protein 1 (*TAX1BP1*) (Figure 6a and Supplementary Figure S13). The augmentation in the expression levels of TNFAIP3, TNIP1, and TAX1BP1 was confirmed in RPE-1 cells treated with EP21-MV (Figure 6b). Furthermore, it was observed that the intra-retinal free ubiquitin level was significantly downregulated in eyes treated with TGF-β2, but was upregulated following treatment with EP21-MV (Figure 6c). Similar results were observed in RPE-1 cells subjected to treatment with a mixture of the six miRNA molecules: levels of free ubiquitin diminished following TNFα treatment, whereas an elevation was noted after the application of the miRNA mixture (Figure 6d).

**Figure 7.**
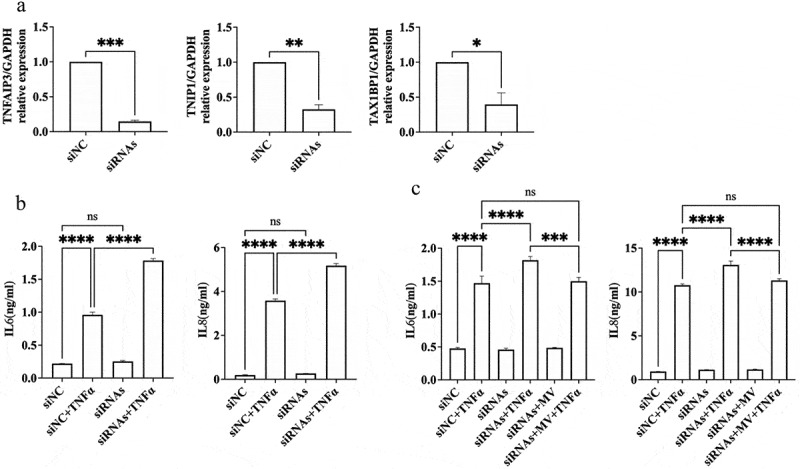
Inhibition of the ubiquitination pathway genes upregulated by *Lactiplantibacillus plantarum* EP21 membrane vesicles exacerbates inflammation. (a) Validation of the gene silencing efficacy of siRNAs targeting TNFAIP3, TNIP1, and TAX1BP1. Mixture of siRNA against TNFAIP3, TNIP1, and TAX1BP1 were transfect into RPE-1 cells and determined the gene silencing efficacy by real-time PCR. T-test was used to evaluate the significant difference between control siRNA (siNC) and siRNA. (b) Pooled siRNAs targeting TNFAIP3, TNIP1, and TAX1BP1 or control siRNA (siNC) were transfected into retinal pigment epithelial cell, RPE-1 and then treated with tumor necrosis factor α (TNFα) for 24 hours. The expression of interleukin 6 (IL6) and IL8 in the culture supernatant was determined by enzyme-linked immunosorbent assay. (c) Pooled siRNAs targeting TNFAIP3, TNIP1, and TAX1BP1 or control siRNA (siNC) were transfected into RPE-1 cell which was treated with EP21 MVs for 24 hours and then treated with TNFα for 24 hours. The expression of IL6 and IL8 in the culture supernatant was determined by enzyme-linked immunosorbent assay. Analysis of variance was applied to examine for significant differences (*p* < 0.05), and Tukey’s multiple comparison tests were used for pairwise comparisons.

We subsequently investigated whether the downregulation of TNFAIP3, TNIP1, and TAX1BP1 expression would influence the anti-inflammatory properties of EP21 MV in RPE-1 cells. The siRNAs targeting TNFAIP3, TNIP1, and TAX1BP1 showed markedly reduced expression levels (Figure 7a). RPE-1 cells that were transfected with a mixture of siRNAs against TNFAIP3, TNIP1, and TAX1BP1 exhibited a significant enhancement in IL6 and IL8 expressions when stimulated by TNFα treatment, in contrast to the siRNA negative control (siNC, Figure 7b). Moreover, treatment with EP21 MV mitigated the TNFα-induced expression levels of IL6 and IL8 induced by the siRNA mixture targeting TNFAIP3, TNIP1, and TAX1BP1 (Figure 7c).

## Discussion

In this study, we demonstrated that *Lactiplantibacillus plantarum* EP21 (EP21) effectively mitigates myopia development by modulating inflammatory pathways within retinal tissues. Both MFD and TGF-β2-induced myopia models confirmed that EP21 administration significantly inhibited axial elongation and refractive error shifts that are typically associated with myopic progression. Notably, EP21 reduced the expression of inflammatory markers, such as NFκB, TNF-α, IL-1β and NLRP3, and this indicated a robust anti-inflammatory response in the retina. Furthermore, EP21-derived MVs crossed the BRB and accumulated within retinal tissues, where they suppressed expression of proinflammatory cytokines. These findings highlight the anti-inflammatory potential of EP21 and its MVs, suggesting that targeting the gut-eye axis through specific probiotics may be a viable strategy to slow or prevent myopia development by attenuating retinal inflammation.

Current research on the gut microbiota’s role in myopia reveals significant associations but is limited by several critical factors that impact the interpretability and generalizability of findings. Most research on the gut microbiota and myopia is cross-sectional, analyzing samples at a single point in time rather than over the disease’s progression.^19,20^ Consequently, it is challenging to establish causal relationships between microbiota changes and myopia progression. Longitudinal studies tracking microbiota composition from early childhood through myopia development would provide stronger evidence of a causal role in disease onset and progression. Environmental factors such as diet, antibiotic exposure, and lifestyle habits can dramatically alter microbiota composition, posing challenges in isolating myopia-specific microbial changes.^26^ Furthermore, the microbiome’s dynamic nature implies that individual microbiota profiles can vary significantly over time, potentially influencing results. Without controlling for these variables, findings may inadvertently capture general microbial dysbiosis rather than a myopia-specific microbial signature.^22^ Our investigation, albeit not explicitly delineating the correlation between dysbiosis and the development of myopia, has yielded substantial evidence suggesting that bacterial membrane vesicles (BMVs) may serve as a potential intermediary in elucidating the mechanisms through which microbiota influence ocular health.

BMVs are the key components that mediate host – microbe interactions by serving as vehicles for communication, immune modulation, and gene transfer. Released by both Gram-positive and Gram-negative bacteria, these nano-sized vesicles (size 20–400 nm) are encased in a lipid bilayer membrane that can protect and deliver a wide array of bioactive molecules, including lipopolysaccharides, proteins, nucleic acids, and other metabolites.^27^ In host – bacteria interactions, BMVs play a significant role by facilitating the transport of bacterial compounds directly into host cells. This process allows bacterial molecules to surpass the limitations of direct bacterial contact to influence distant host cellular pathways and immune responses.^28^ BMVs are increasingly being studied for their biomedical potential, particularly as platforms for drug delivery and vaccines. The BMVs’ natural immunogenicity makes them ideal self-adjuvant nanovaccines, and their structure allows targeted delivery of therapeutic molecules to improve disease outcomes in infections, autoimmune disorders, and cancer.^29,30^ MVs derived from *Lactiplantibacillus plantarum* and *Lactobacillus reuteri* can downregulate proinflammatory cytokines such as TNF-α, IL-6, and IL-1β, which are the key markers in inflammatory responses.^31,32^ By reducing these cytokines, Lactobacillus MVs help mitigate inflammatory conditions such as colitis and lipopolysaccharide induced intestinal inflammation. Furthermore, *Lactobacillus reuteri* MVs reportedly promotes anti-inflammatory cytokines, including IL-10 and TGF-β, thereby enhancing immune homeostasis and modulating the host’s immune response toward tolerance rather than inflammation.^32^ *Lactobacillus sakei* MVs contribute to gut health by promoting the production of immunoglobulin A, which reinforces the mucosal barrier against pathogens and further decreases inflammation.^33^ Although these studies indicate the beneficial effects of MVs, the specific components responsible in the MVs for their biological effects remain unreported. We documented that EP21 MVs infiltrate the BRB and the BBB, and thereby influencing their biological activity and miRNAs contained within the EP21 MVs. This modulated the expression of the NFκB signaling pathway and the ubiquitin-proteasome system, and ultimately led to the attenuation of inflammatory responses within the ocular environment.

Two primary hypotheses exist concerning how probiotics modulate biological activities in mammalian cells or tissues: through metabolites produced by probiotics or through toll-like receptor signaling pathways influenced by MVs.^34^ EP21 administration elevates the serum concentration of tryptophan metabolites such as indole-3-propionic acid and indolelactic acid (Supplementary Figure S14A). Indole-3-propionic acid reportedly activates the aryl hydrocarbon receptor, and thereby mitigates intraretinal inflammation and offers protection against retinal ganglion cell apoptosis in glaucoma models.^35^ Moreover, indolelactic acid interacts with the aryl hydrocarbon receptor to downregulate proinflammatory pathways, including NFκB and hypoxia-inducible factors signaling, and reduce the production of inflammatory cytokines, such as chemokine (C-C motif) ligand 2/7 and IL-8, to ameliorate intestinal epithelial barrier damage in mice.^36^ Besides modulating tryptophan metabolites (Supplementary Figure S14A), EP21 influences the serum levels of SCFAs, including acetic acid and isovaleric acid (Supplementary Figure S14B). However, there was no statistically significant difference in the concentrations of propionic acid, butyric acid, isobutyric acid, and 2-methylbutyric acid between control and EP21-fed mice (Supplementary Figure S14B). Due to an unknown mechanism, the administration of EP21 significantly increases the concentration of acetic acid in serum while slightly downregulating the concentration of isovaleric acid. The production of SCFAs is influenced by a variety of stimuli, including feeding time and the quantity of bacteria administered.^37^ The total SCFA concentration is augmented because of the elevated levels of acetic acid in serum (Supplementary Figure S14B). Consequently, EP21 has modified the concentrations of SCFA and tryptophan metabolites in serum as well as in the secreted MVs to inhibit myopia progression. The etiologic significance of SCFAs and tryptophan metabolites in myopia remains unclear. SCFAs function as histone deacetylase inhibitors, inducing changes in gene expression that subsequently influence cell membrane properties. For example, SCFAs enhance Notch signaling, which promotes enterocyte differentiation and decreases intestinal permeability, indicating their potential role in modifying membrane characteristics.^38^ Additionally, SCFAs strengthen intestinal barrier function and reduce permeability by increasing transepithelial electrical resistance and fostering enterocyte differentiation, indicating that SCFAs influence membrane fluidity.^39^ Moreover, SCFAs protect intestinal cells against LPS-induced barrier disruption by preserving essential tight junction proteins, such as ZO-1 and occludin, thereby maintaining membrane integrity and fluidity.^40^ Despite the lack of direct evidence in the literature, the SCFA-based alteration of membrane properties could potentially affect MV processing and absorption by host cells or other bacteria in the gut. These combined actions could create a coordinated response whereby SCFAs prime host cells to become more receptive to regulatory molecules that are delivered via bacterial MVs.

Regulating free ubiquitin levels is critical for cellular health, particularly in tissues that are prone to oxidative stress and inflammation, such as the retina. Free ubiquitin levels are managed through synthesis, recycling, and degradation pathways. Ubiquitin is encoded by monomeric ubiquitin-ribosomal fusion genes (ubiquitin A-52 residue ribosomal protein fusion product 1 and ribosomal protein S27a) and polyubiquitin genes (ubiquitin B and ubiquitin C). During stress or heightened protein turnover, these genes are upregulated to supply more free ubiquitin when its demand increases. However, disruptions in ubiquitin B or ubiquitin C can lower free ubiquitin levels, weakening the cellular response to stress.^41,42^ Recycling is another major mechanism for maintaining free ubiquitin. Deubiquitinating enzymes cleave ubiquitin from conjugated proteins, replenishing the free ubiquitin pool. Over 100 deubiquitinating enzymes in humans specifically recognize and release ubiquitin from substrates, making this process highly adaptable to changes in cellular demand.^43,44^ In addition, cellular signaling pathways can be activated to modulate deubiquitinating enzyme activity or ubiquitin synthesis in response to environmental stress. Conversely, ubiquitin itself can undergo degradation under conditions of excess free ubiquitin to avoid cellular imbalance. For the eye, ubiquitin’s regulation helps prevent retinal degeneration – a condition seen in ubiquitin B knockout models, which show retinal layer thinning and photoreceptor loss.^45^ These effects highlight how the maintenance of a free ubiquitin pool is essential for ensuring the resilience of retinal cells against inflammation-induced proteotoxic stress. To manage ubiquitin levels, the balance between ubiquitin synthesis and recycling by deubiquitinating enzymes is vital.^44,46^ Overall, stabilizing free ubiquitin can offer neuroprotective benefits, particularly in the eye, by enabling continuous proteasomal function and mitigating inflammatory responses. TNFAIP3, also known as A20, is a ubiquitin-editing enzyme that regulates inflammation by removing ubiquitin chains from specific substrates. This action is crucial for controlling the activation of NF-κB, a transcription factor that mediates inflammatory responses.^47,48^ TNIP1 plays a significant role in inhibiting inflammatory signaling pathways, interacts with ubiquitin through its AHD1-UBAN domain, which subsequently inhibits NF-κB signaling. TNIP1 deficiency can lead to hyper-responsive inflammatory signaling, as seen in keratinocytes, where it affects wound healing and cell viability.^49^ The relationship between TAX1BP1 and free ubiquitin is intricately linked to autophagy, particularly in the context of stress-induced protein aggregate clearance.^50^ The recruitment of TAX1BP1 to ubiquitin-rich sites is enhanced by an increased ubiquitin load, which suggests that the presence of free ubiquitin or ubiquitin chains can influence the localization and activity of TAX1BP1.^51^ EP21-MVs increase the concentrations of TNFAIP3, TINP1, and TAX1BP1, and thereby modify the ubiquitin-proteasome pathway and regulate the inflammatory response within the ocular environment, which consequently contributes to the suppression of myopia advancement.

EP21 inhibits myopia development through a multifaceted mechanism that centers on its secreted MVs, immunomodulatory RNAs, and systemic metabolic effects. These molecular mechanisms converge to suppress retinal inflammation and structural remodeling associated with myopia. Upon oral administration, EP21 releases MVs that systemically circulate and penetrate the BRB. Once localized to the retinal outer segment, these vesicles deliver bioactive contents including miRNAs that directly modulate inflammatory signaling. One central pathway targeted is NFκB, a transcriptional hub that, when activated by TGF-β, IL-1β or TNFα, stimulates downstream genes including MMP2. MMP2 mediates extracellular matrix degradation, promoting scleral thinning and axial elongation – hallmarks of myopia. EP21-MVs inhibit NFκB phosphorylation and nuclear translocation, thereby downregulating MMP2 and preventing excessive remodeling. Simultaneously, collagen type I (COL1A1) expression is enhanced, reinforcing scleral integrity. In addition, this inhibition results in reduced expression of TNFα, IL-1β, and NLRP3, effectively curtailing inflammasome activation – a known driver of myopic pathology. In parallel, EP21-MVs enhance the expression of anti-inflammatory IL-10. miRNAs within EP21-MVs target transcripts involved in the NFκB pathway and inflammation-related ubiquitin signaling. The vesicles upregulate ubiquitination-regulatory genes such as TNFAIP3, TNIP1, and TAX1BP1, which act to degrade or deubiquitinate proinflammatory signaling intermediates, thereby attenuating downstream cytokine production. This contributes to proteostasis and prevents inflammation-induced tissue remodeling. Furthermore, EP21 alters systemic metabolites including short-chain fatty acids and tryptophan derivatives, which suppress inflammatory signaling. These gut-derived metabolic signals reinforce anti-inflammatory responses and possibly influence MV uptake or stability at the ocular level. In summary, EP21 inhibits myopia by (1) delivering anti-inflammatory miRNAs via membrane vesicles to retinal cells, (2) modulating NFκB and NLRP3 inflammasome activation, (3) altering MMP2-mediated tissue remodeling, (4) enhancing anti-inflammatory ubiquitination pathways, and (5) modifying systemic metabolites to inhibit inflammatory signaling (Supplementary Figure S15). These findings indicate a gut – eye axis where probiotics and their vesicles could modulate ocular health and thereby constitute a promising direction for managing the global myopia epidemic through noninvasive, biological interventions.

## Supplementary Material

Supplementary Data 1216.docx

## Data Availability

*Lactiplantibacillus Plantarum* EP21 generated in this study have been deposited to Food Industry Research and Development Institute, HsinChu, Taiwan: accession number BCRC911210. RNA-seq data have been deposited in Sequence Read Archive: PRJNA1187797 and PRJNA1187848 and are publicly available as of the date of publication. Original western blot images have been deposited at Mendeley at [DOI: 10.17632/3m28wjgtdh.1] and are publicly available as of the date of publication. Original Ingenuity Pathway Analysis data have been deposited at Mendeley at [DOI: 10.17632/38wctrkx3h.1] and are publicly available as of the date of publication. Any additional information required to reanalyze the data reported in this paper is available from the lead contact upon request.
