# Supplementary material for: Harnessing Lactiplantibacillus plantarum EP21 and its membrane vesicles to inhibit myopia development
Source: Gut Microbes. 2025 Aug 1;17(1):2534677. doi: 10.1080/19490976.2025.2534677 (PMC12320841; doi:10.1080/19490976.2025.2534677)
Supplement: Supplementary Data 1216.docx [file KGMI_A_2534677_SM3825.docx]

**Supplementary Data**

***Bacterial strain identification***

16S rDNA of *Lactiplantibacillus plantarum* EP21 was amplified by primers 8F (5′-AGAGTTTGATCMTGGCTCAG-3′) and 1492R (5'-CGGTTACCTTGTTACGACTT-3'

) by way of colony polymerase chain reaction (colony PCR) under the following conditions: denaturation at 94°C for 2 min, followed by 30 cycles at 94°C for 1 min, 58°C for 1 min, and 72°C for 2 min, with a final 10 min of extension at 72°C. PCR product was purified and sequenced on a DNA sequencer (Model: ABI 3730XL) at the Genomics BioScience and Technology Co., Ltd. (Taiwan). EP21 was confirmed to be a *Lactiplantibacillus plantarum* species by searching its 16S rDNA sequence with the Basic Local Alignment Search Tool (BLAST) web service of the National Center for Biotechnology Information (NCBI). The result was confirmed to be about 99% similar to submitted sequences of *Lactiplantibacillus plantarum* in the NCBI nucleotide database. Live *Lactiplantibacillus plantarum* EP21 was deposited in the Food Industry Research and Development Institute (Taiwan; accession number BCRC911210).

***RNase Protection Assay (RPA)***

Twenty μg total RNA purified from membrane vesicles, *Lactiplantibacillus plantarum* EP21 and methicillin-resistant *Staphylococcus aureus* was hybridized with 0.2 μM antisense probes of NovelmiRNA (Supplementary Table 2) in hybridization buffer (2 M NaCl, 5 mM EDTA, 200 mM PIPES) at 45℃ for 18 hr. RPA products were digested by RNase digestion buffer (300 mM NaCl, 5 mM EDTA, 10 mM Tris-HCl) and precipitated by 3M NaOAC. 15% native polyacrylamide gel (40% (19:1) bis-acrylamide, 10x TBE buffer, DEPC treated H_2_O) and low range DNA Ladder (Cat. # N0558; NEW ENGLAND Biolabs, USA) were used in this experiment. Gel electrophoresis was done with a 1x TBE buffer. After the orange G dye got close to the bottom of the gel, approximately at 5 mm, the gel was stained with SYBR™ Safe stain (Cat. # S33102; Thermo Fisher Scientific, USA), gently shaking for 30 min. The images were taken by blue-light transilluminator (Cat. # BK001).

***Primary human retinal pigment epithelial cell***

Two different primary retinal pigment epithelial cells were purchased from ScienCell Research Laboratories (Cat. # 6540, USA) and Innoprot Inc (Cat. # P10873, Spain). hRPEpiC cell (human primary retinal epithelial cell) was purchased from ScienCell Research Laboratories (ScienCell, USA). The cells were maintained in epithelial cell medium from ScienCell Research Laboratories (Cat. #4101, USA) and Innoprot Inc (Cat. # P60106, Spain) and followed the culture protocols provided by the manufacturers. Cells were seeded in 6-well plates (3$\times$10^5^ cells/well) overnight before treatments. The plates were pre-coated with poly-lysine (2 μg/cm^2^) (ScienCell, Cat. # 0413, USA) as the manufacturer recommended.

***Whole Transcriptome Sequencing Analysis (WTS) and Ingenuity Pathway Analysis (IPA)***

Primary RPE cells were seeded at 3$\times$10^5^ cells/well in 6-well plates for 16 hr. EP21 MVs (2$\times$10^6^ particles/well) were applied to treat cells for 2 hr. Total RNA was extracted by the Trizol reagent (Qiagen; Cat. # 79306, USA). WTS was performed by Azenta Life Sciences Co (USA). Novel miRNAs sequences were predicted by the hairpin structure of precursors and determined by the secondary structure via miRDeep2 (V2_0_0_8). Differential expression analysis (DEA) of mRNAs was used to analyze the regulatory networks between control group (NC) and MV-treated group (MV) via the IPA.

***Serum sample preparation procedures***

*Lactiplantibacillus Plantarum* EP21 was orally fed C57BL/6 mice for 3 weeks. Sera were collected to determine the concentration of short chain fatty acid (SCFA) and tryptophan metabolite by liquid chromatograph coupled with an Agilent 1290 Infinity II UHPLC coupled with an Agilent 6495C triple quadrupole mass spectrometer (Agilent Technologies, Santa Clara, CA). For short chain fatty acids, we determined the concentrations of acetic acid., propionic acid, butyric acid, isobutyric acid, 2-methylbutyric acid and isovaleric acid. For tryptophan metabolites, we determined the concentrations of tryptophan, indole-3-acetic acid, indole-3-propionic acid, indolelactic acid and hippuric acid.

Forty microliters of the serum samples were mixed with 160 μL of MeOH for protein precipitation. The mixture was vortexed using a Geno/Grinder 2010 (SPEX, Metuchen, NJ, USA) at 1000 rpm for 5 minutes. Subsequently, the sample was centrifuged at 18,000 rcf for 10 minutes at 4 °C. Sixty microliters of the supernatant was collected and spiked with 30 μL of 0.2M 3-Nitrophenylhydrazine hydrochloride (3NPH·HCl) and 30 μL of 0.12M N-(3-dimethylaminopropyl)-N-ethylcarbodiimide (EDC·HCl) for derivatization. The mixture was allowed to react at 40 °C for 20 minutes. Afterward, the sample was cooled on ice for 3 minutes, followed by the addition of 30 μL of 13C6-Nitrophenylhydrazine hydrochloride (^13^C_6_-3NPH·HCl) 13C6-3NPH internal standard. Finally, the sample was filtered through a 0.22 μm Minisart RC 4 cellulose membrane and analyzed by Ultra-high performance liquid chromatography-MS/MS (UHPLC-MS/MS)

Supplementary Table 1. Quantitative real-time PCR primers used in this study

| Gene | Forward (5′–3′) | Reverse (5′–3′) | Accession Number |
| --- | --- | --- | --- |
| *hTAB3* | GAAAGGGCTCGGCAACAGG | AATTCCAGAGGGCGTGGTGT | NM_001399870.1 |
| *hTNFA* | GGAGAAGGGTGACCGACTCA | CCTCACAGGGCAATGATCCC | NM_000594.4 |
| *hRELA* | GTATCTGTGCTCCTCTCGCC | TGGCCCCTATGTGGAGATCA | NM_001404662.1 |
| *hCASP1* | CCACACTCCCGACCATACAC | TGCCCACCACTGAAAGAGTG | NM_001257119.3 |
| *hIL1B* | GTGGTGGTCGGAGATTCGTA | TCTTTGAAGCTGATGGCCCTA | NM_000576.3 |
| *hIL6* | TACCTAGAGTACCTCCAGAACAGA | TGCAGGAACTGGATCAGGACT | NM_001371096.1 |
| *hTNFAIP3* | GGTGTCGAGAAGTCCGGAAG | GAACAGCGCCTTCCTCAGTA | NM_001270507.2 |
| *hTNIP1* | TCAGGAGTGACCACTTCAAATTCA | ATGCCCCAGTGACAAGCC | XM_054351427.1 |
| *hTAX1BP1* | TTACCTTCCTAATGCACACCTGG | ATGTTCAGGCATAGGGGACCA | NM_001206902.2 |

Supplementary table 2. RNase Protection Assay (RPA) probes used in this study

| Entity | Novel miRNA | Sequence |
| --- | --- | --- |
| Probe 1 | NovelmiRNA-284 | ATCCAGTGACCCCAGTCTCGGT |
| Probe 2 | NovelmiRNA-487 | AGGGGTCATGGGGGTGGT |
| Probe 3 | NovelmiRNA-491 | GGGACAGGAGGTCCTCGAGTG |
| Probe 4 | NovelmiRNA-213 | CATCCACCGGACTGACCGT |
| Probe 5 | NovelmiRNA-139 | GGGTATGAAAGTGGGGAGAGA |
| Probe 6 | NovelmiRNA-85 | CAGGGACAAGTCCGCGGT |

 
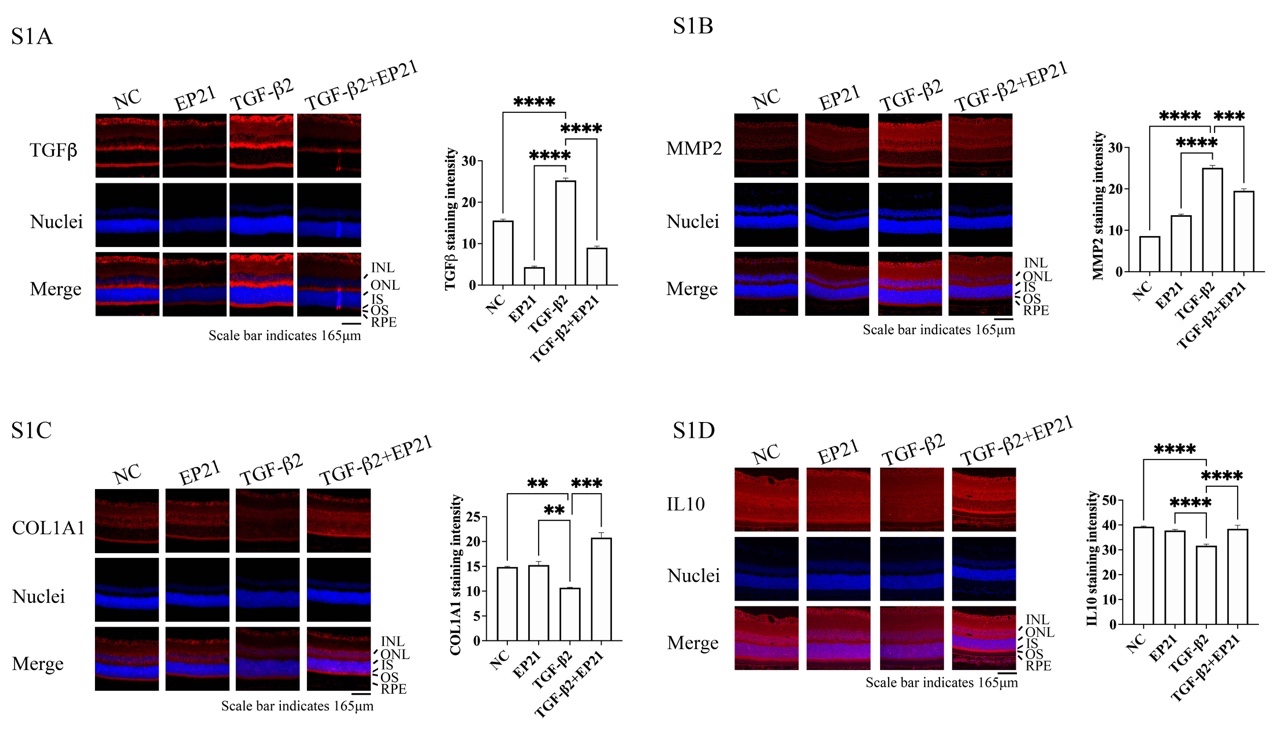


Supplementary Figure 1: Probiotic treatment suppresses the myopia development and inflammatory reactions in the eye. Immunofluorescence staining of transforming growth factor-β (TGFβ) (A), matrix metalloproteinase-2 (MMP2) (B), collagen type I alpha 1 (COL1A1) (C), and interleukin 10 (IL10) (D) expression in the eyes of NC, EP21, TGF-β2 and TGF-β2 + EP21-treated rats. Relative expression levels were determined using Image J software. Results are shown as mean ± SD. The scale bar indicates 165 μm and 200× magnification for IF staining images. Analysis of variance was applied to examine for significant differences (P < 0.05), and Tukey’s multiple comparison tests were used for pairwise comparisons. NC: negative control. INL: inner nuclear layer; ONL: outer nuclear layer; IS: inner segment; OS: outer segment; RPE: retinal pigment epithelium.

 
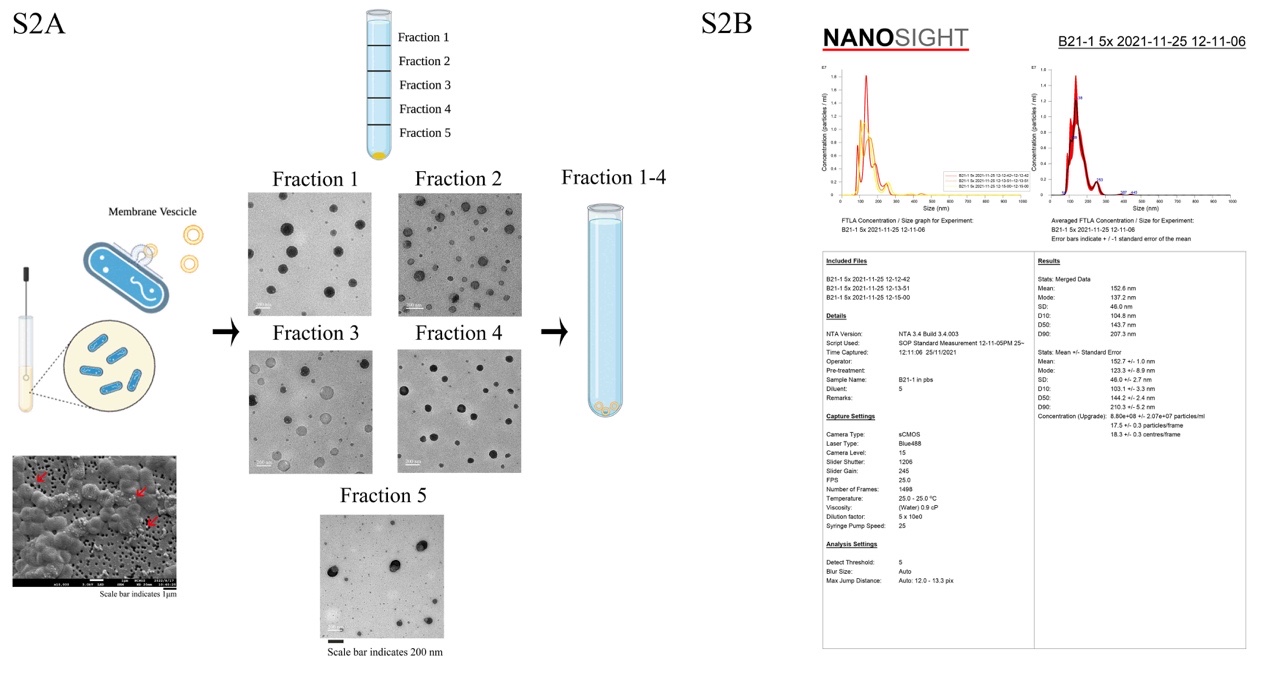


Supplementary Figure 2: Isolation and verification of membrane vesicles (MVs) secreted by *Lactiplantibacillus plantarum* EP21 (EP21).

(A) EP21 MVs emerged spontaneously from their cellular matrix during the exponential growth phase and were subsequently imaged utilizing Cryo-SEM. The MVs were isolated through the process of ultracentrifugation and further purified via an OptiPrep^TM^ concentration gradient, resulting in the division into five distinct fractions. Each of these fractions underwent analysis through Transmission Electron Microscopy (TEM). The MVs were collected from fractions one to four, owing to the superior integrity of the MVs in comparison to that observed in fraction five. Scale bars in the Cryo-SEM image indicate 1 µm. Scale bars in the TEM image indicate 200 nm. (B) Particle concentration and size distribution of EP21 MVs were measured by nanoparticle tracking analysis (Malvern NanoSight NS300).


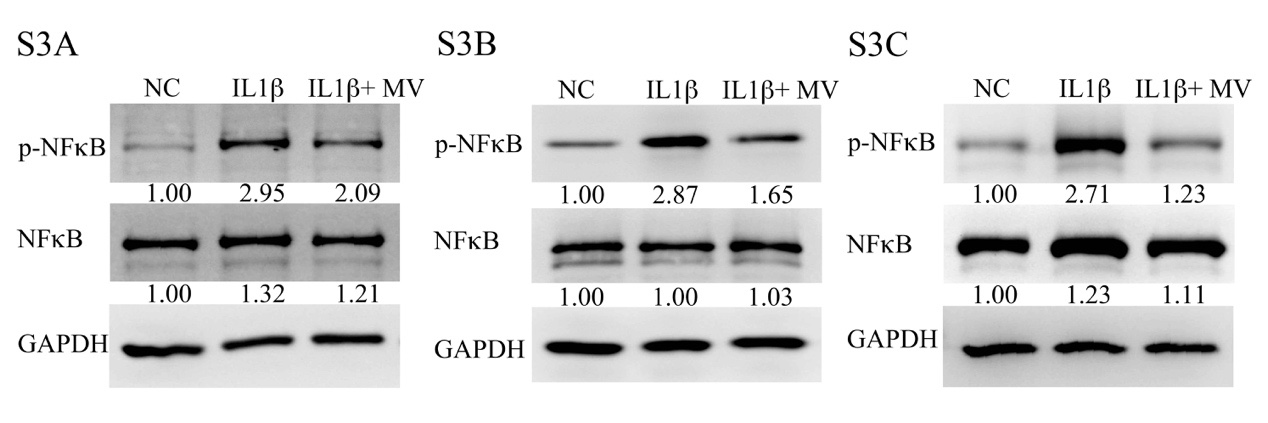


Supplementary Figure 3: *Lactiplantibacillus plantarum* EP21 membrane vecisles inhibit interleukin (IL)-1β induced nuclear factor κB expression and activation. (A) ARPE-19 cells; (B) human retinal pigment epithelial cell from ScienCell, USA (C) human retinal pigment epithelial cell from Innoprot, Spain. Image J software was used to determine the expression levels. Relative expression levels of p-NF-κB and NF-κB (below the lanes) were normalized to glyceraldehyde-3-phosphate dehydrogenase (GAPDH) and the expression levels in lane negative control (NC) are designated as 1.


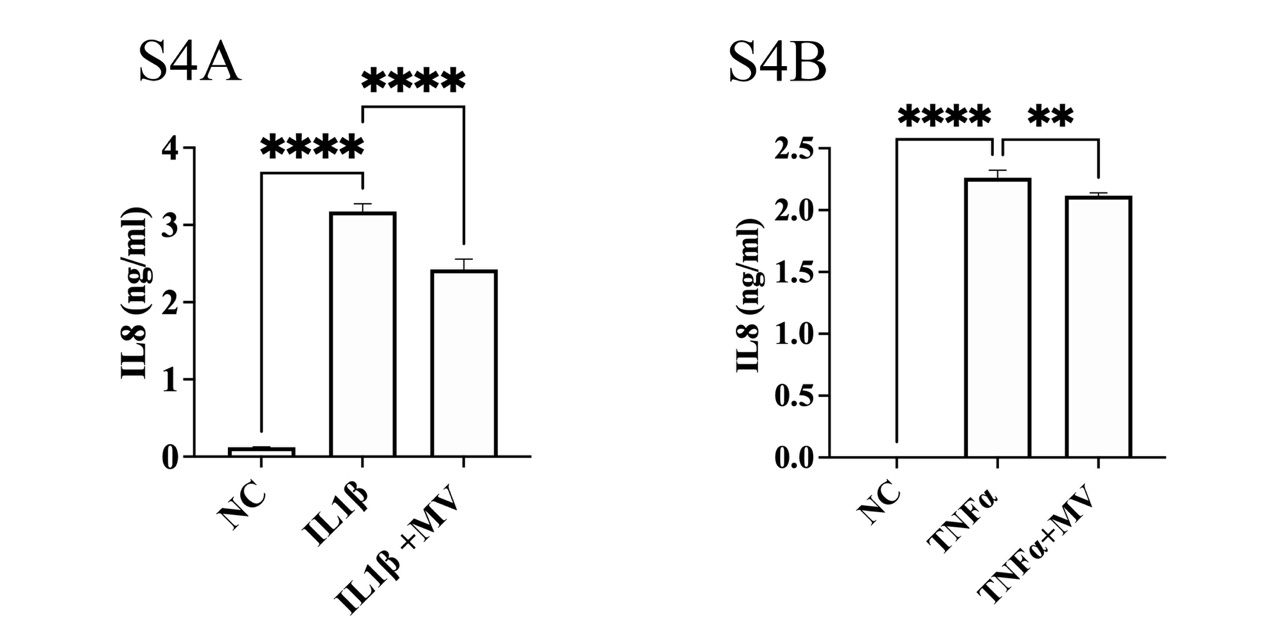


Supplementary Figure 4: *Lactiplantibacillus plantarum* EP21 membrane vesicles (MV) inhibit IL1β (A) and TNFα (B) induced IL-8 expression in RPE-1 cells. Cells were treated with phosphate buffered saline (NC), IL1β (or TNFα), or IL1β (or TNFα) + MV for 24 hours. The IL8 concentration was then determined by enzyme-linked immunosorbent assay. Results are shown as mean ± SD. Analysis of variance was applied to examine for significant differences (P < 0.05), and Tukey’s multiple comparison tests were used for pairwise comparisons.


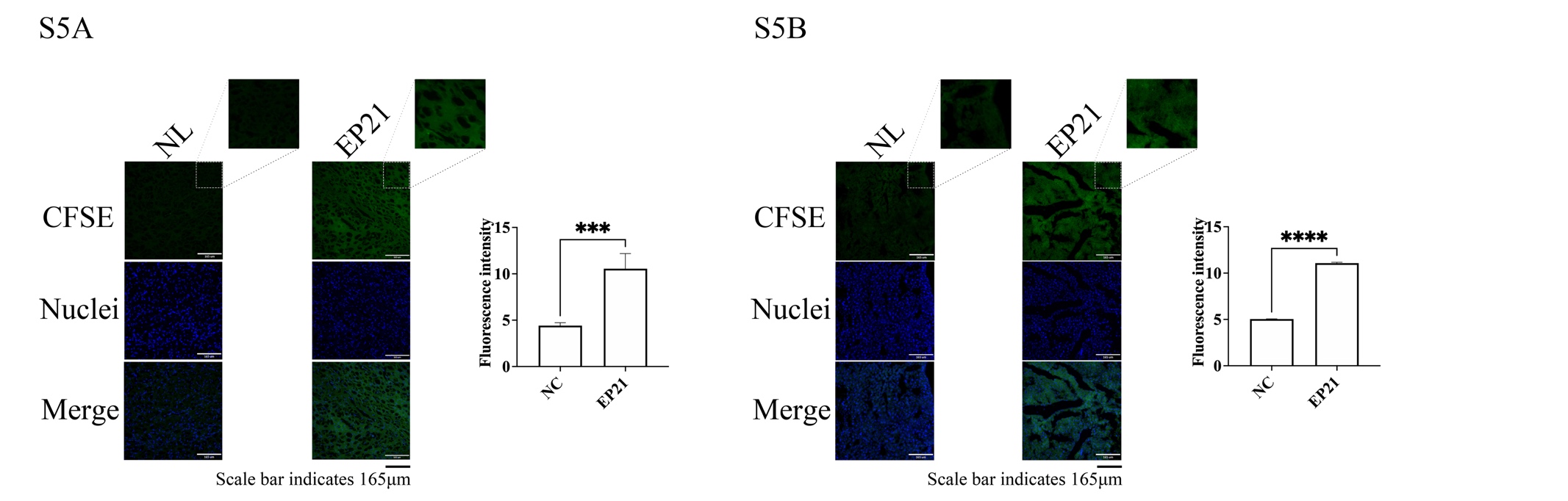


Supplementary Figure 5: Membrane vesicles secreted by *Lactiplantibacillus plantarum* EP21 (MV) pass across blood-brain-barrier (BBB). EP21 was stained with or without carboxifluorescein diacetate succinimidyl ester (CFSE) and orally fed C57BL/6 mice for 7 days. Brain (A) and liver (B) were collected to determine the fluorescence intensities. Results are shown as mean ± SD. Relative expression levels were determined using Image J software. T-test was used to evaluate the significant difference between non-labeled (NL) and EP21.


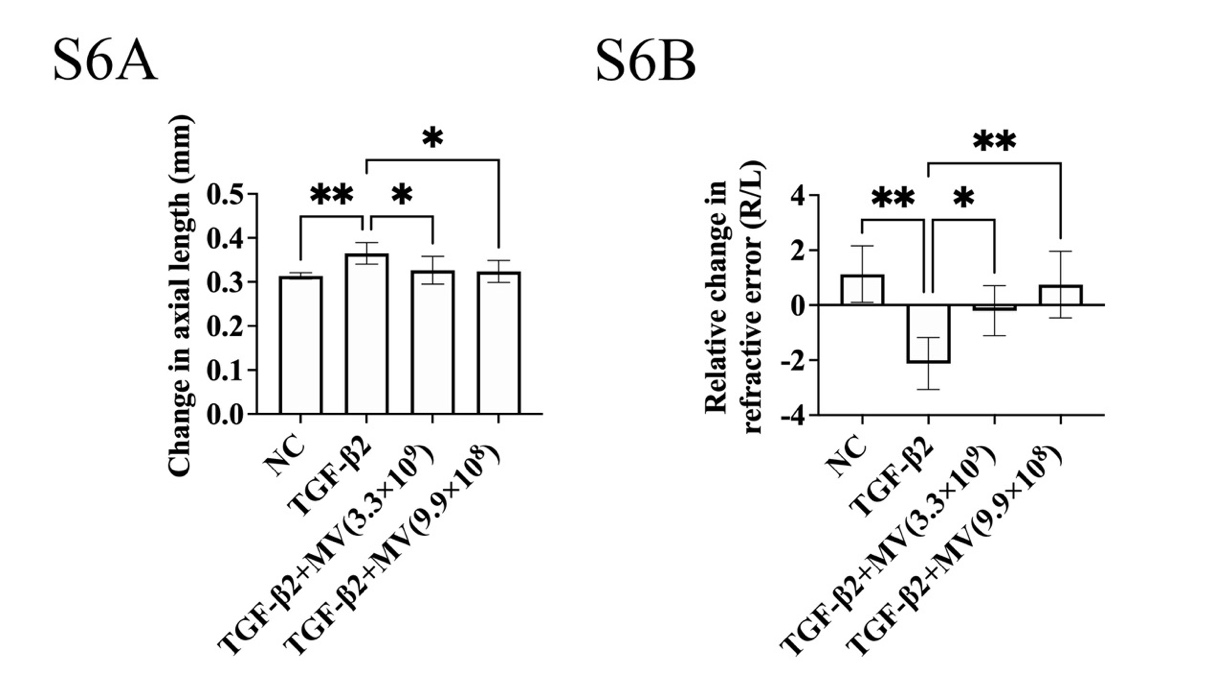


Supplementary Figure 6: *Lactiplantibacillus plantarum* EP21 membrane vesicles (EP21 MVs) treatment suppresses the development of myopia. Myopia was induced by subjunctival injection of transforming growth factor-β2 (TGF-β2). (A) EP21 MVs were reconstituted in a balanced salt solution and subsequently administered directly to the ocular surface (20 μL) every day for 21 days. The specified quantity reflected the cumulative number of MVs administered to the ocular region throughout the experimental procedures. The administration of membrane vesicles effectively inhibited the elongation of axial length induced by the subconjunctival injection of TGF-β2. (B) EP21 MVs inhibited the down-regulation of refractive error induced by TGF-β2. The relative change in refractive error of the right eye was standardized to the refractive error of the left eye, which remained untreated. Results are shown as mean ± SD. Analysis of variance was applied to examine for significant differences (P < 0.05), and Tukey’s multiple comparison tests were used for pairwise comparisons.

 
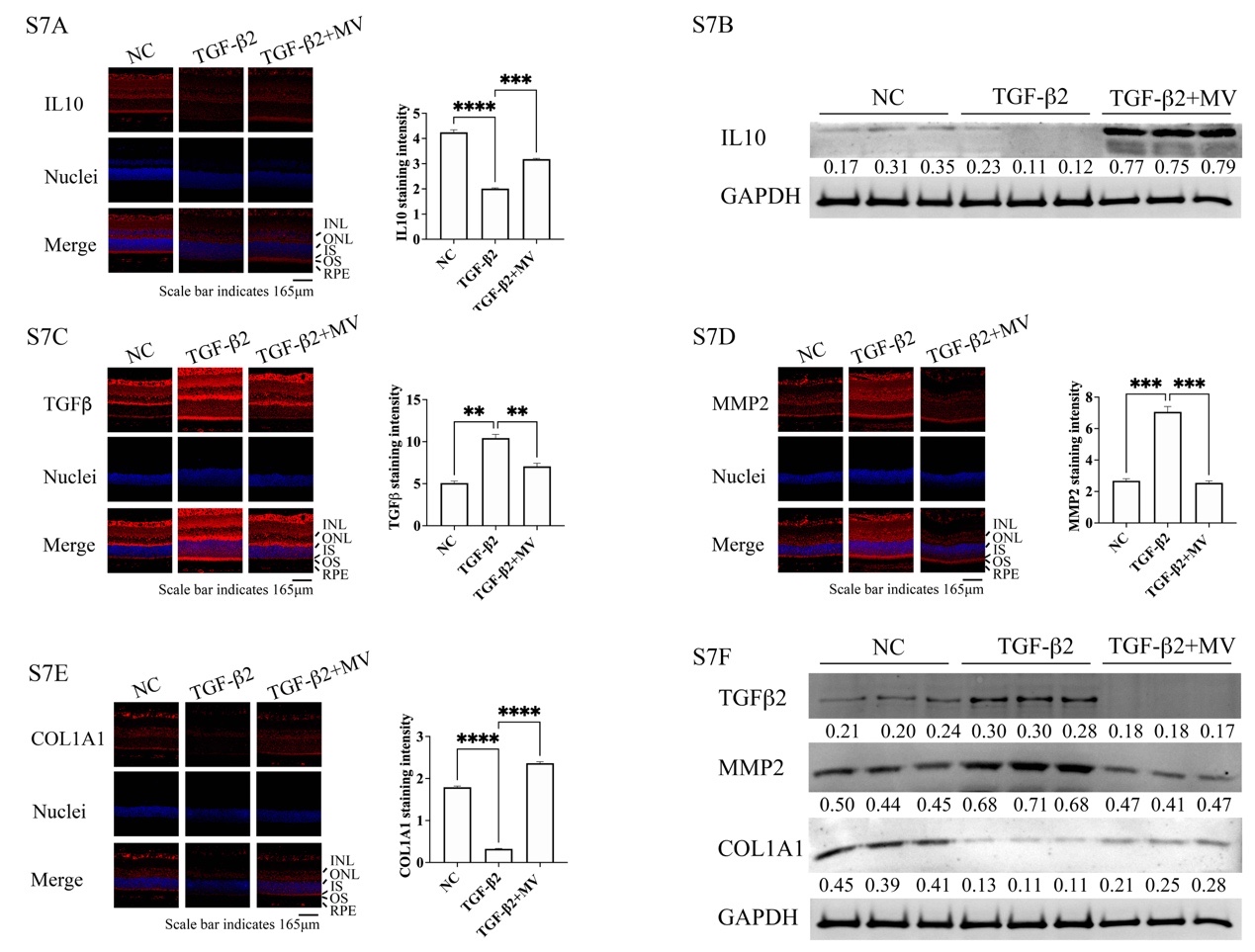


Supplementary Figure 7: *Lactiplantibacillus plantarum* EP21 membrane vesicles (EP21 MVs) treatment suppresses the development of myopia by lowering the inflammatory responses. Myopia was induced by subjunctival injection of transforming growth factor-β2 (TGF-β2). (A) Immunofluorescence staining (A) and western blot (B) analysis of interleukin 10 (IL-10) expression in the retina of NC, TGF-β2 and TGF-β2 + MV-treated rats. Immunofluorescence staining analysis of transforming growth factor-β (TGF-β) (C), matrix metalloproteinase-2 (MMP-2) (D) and collagen type I alpha 1 (COL1A1) (E) expression in the retina of NC, T2 and T2 + MV-treated rats. (F) Western blot analysis of the expression level of TGF-β, MMP2 and COL1A1 in the retina of NC, TGF-β2 and TGF-β2 + MV-treated rats. The scale bar indicates 165 μm and 200× magnification for IF staining images. Results are shown as mean ± SD. Analysis of variance was applied to examine for significant differences (P < 0.05), and Tukey’s multiple comparison tests were used for pairwise comparisons. Relative expression levels were determined using Image J software. NC: negative control. INL: inner nuclear layer; ONL: outer nuclear layer; IS: inner segment; OS: outer segment; RPE: retinal pigment epithelium. For western blot, relative expression levels (below the lanes) were normalized to glyceraldehyde-3-phosphate dehydrogenase (GAPDH).


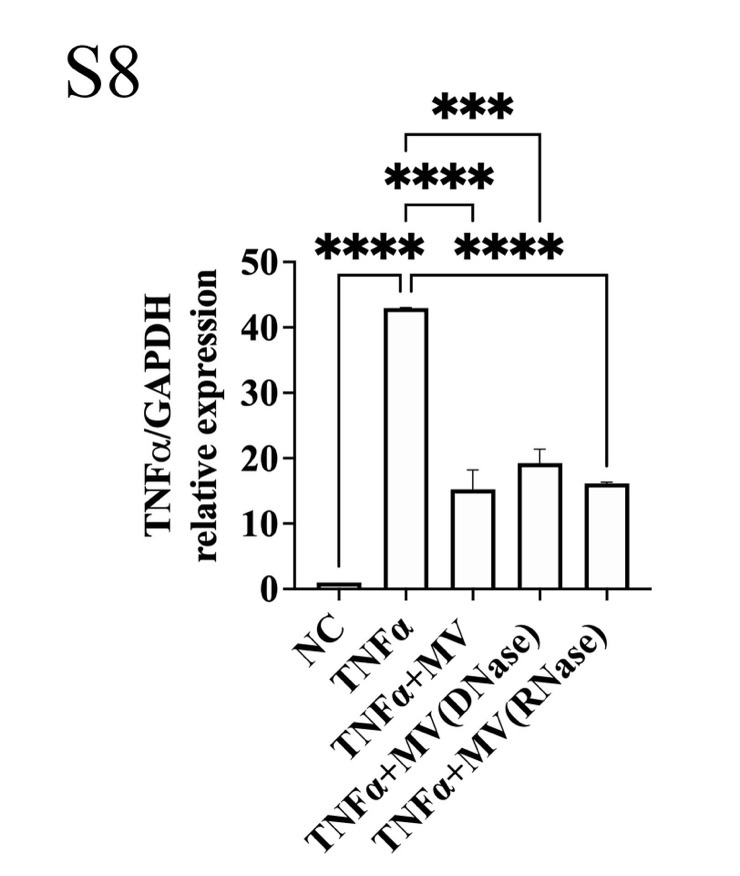


Supplementary Figure 8: *Lactiplantibacillus plantarum* EP21 membrane vesicles (EP21 MVs) inhibited inflammatory reactions in the human microglial cell. Intact EP21 MVs were treated with DNase I or RNase A to remove DNA or RNA binding to the surface of EP21 MVs. HMC-3 cells were treated with phosphate buffered saline (NC), tumor necrosis factor α (TNFα), MV, DNA I or RNase A treated MVs for 12 hrs. EP21 MVs inhibited the expression of TNFα determined by quantitative realtime polymerase chain reaction. Results are shown as mean ± SD. Relative expression levels were normalized to glyceraldehyde-3-phosphate dehydrogenase (GAPDH). Analysis of variance was applied to examine for significant differences (P < 0.05), and Tukey’s multiple comparison tests were used for pairwise comparisons.


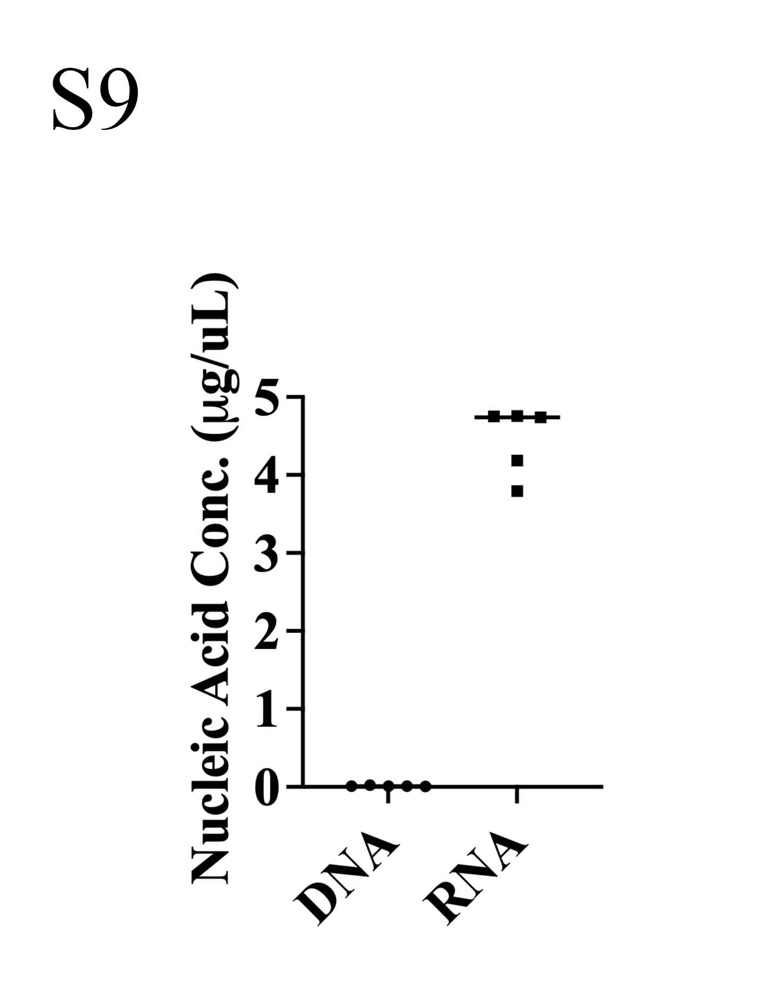


Supplementary Figure 9: Nucleic acids extracted from *Lactiplantibacillus plantarum* EP21 membrane vesicles from 5 different batches. DNA or RNA concentration was determined by a Nanodrop spectrometry.


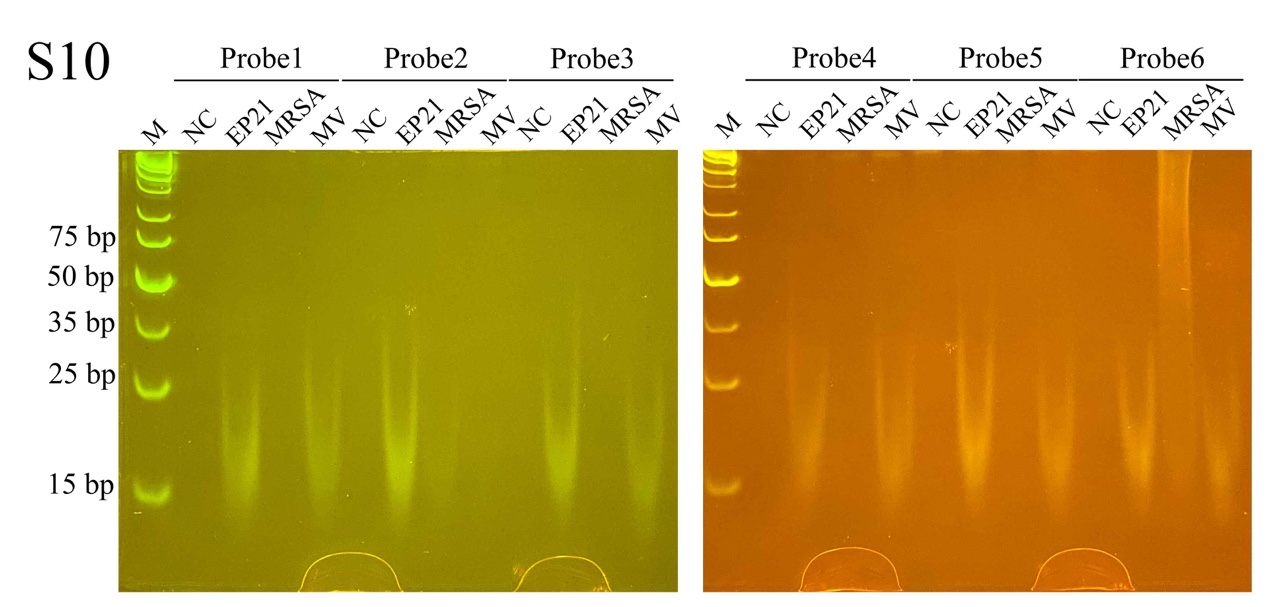


Supplementary Figure 10: The presence of miRNA in the EP21 MVs detected by RNase protection assay analysis. Antisense oligos against NovelmiRNA-284 (probe 1), 481 (probe 2), 491 (probe 3), 213 (probe 4), 139 (probe 5) and 85 (probe 6) were used to hybridize the RNAs extracted from EP21, methicillin-resistant Staphylococcus aureus (MRSA) or EP21 MVs.  A native polyacrylamide gel electrophoresis was used to separate the hybridized DNA-RNA duplex.


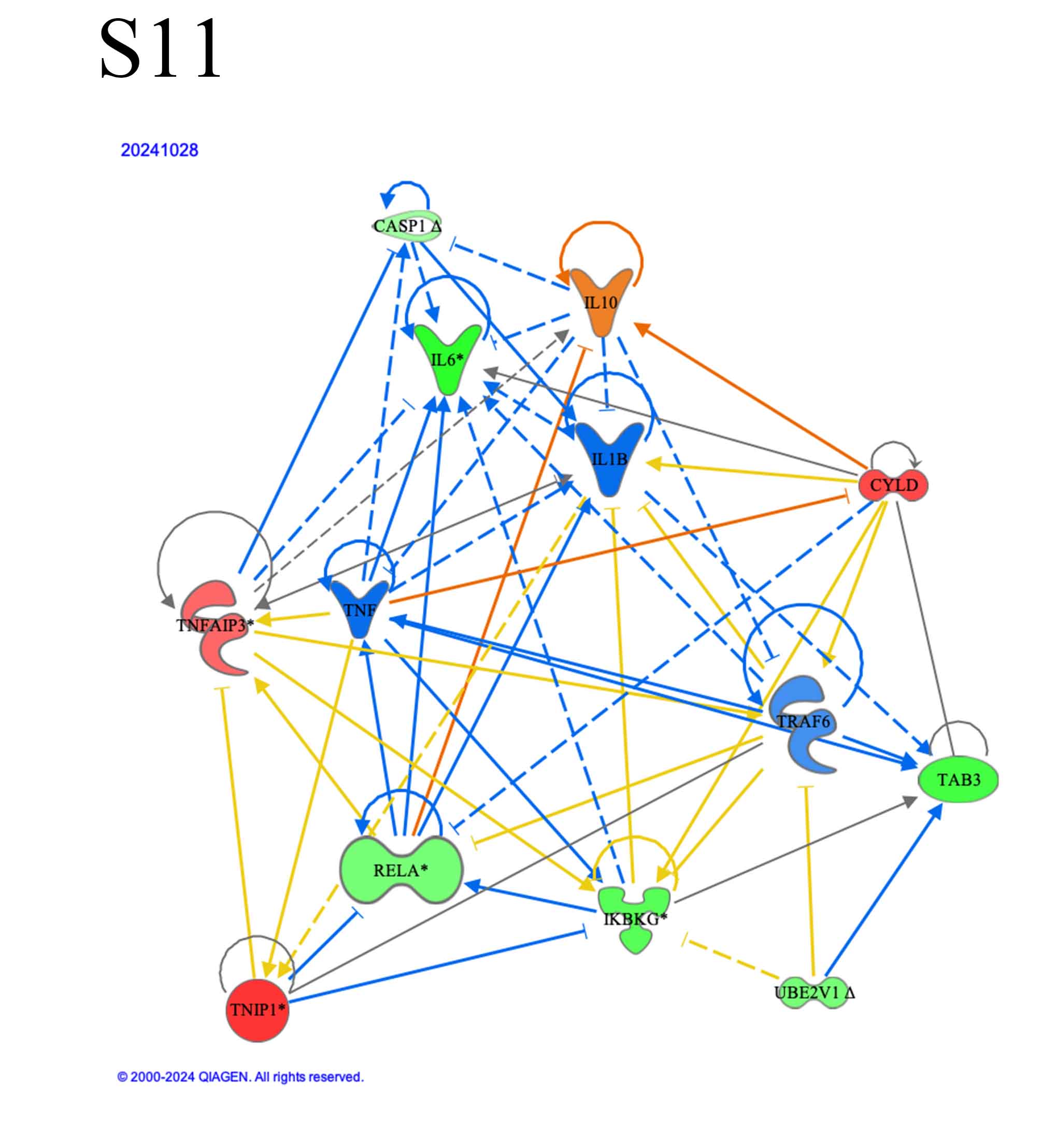


Supplementary Figure 11: Differentially expressed gene (DEGs) expressions in primary retinal pigment epithelial cells (from ScienCell, USA) treated by *Lactiplantibacillus plantarum* EP21 membrane vesicles (EP21 MVs). Ingenuity pathway analysis identified the interaction between the inflammatory pathway genes and ubiquitin pathway genes in retinal pigment epithelial cells.


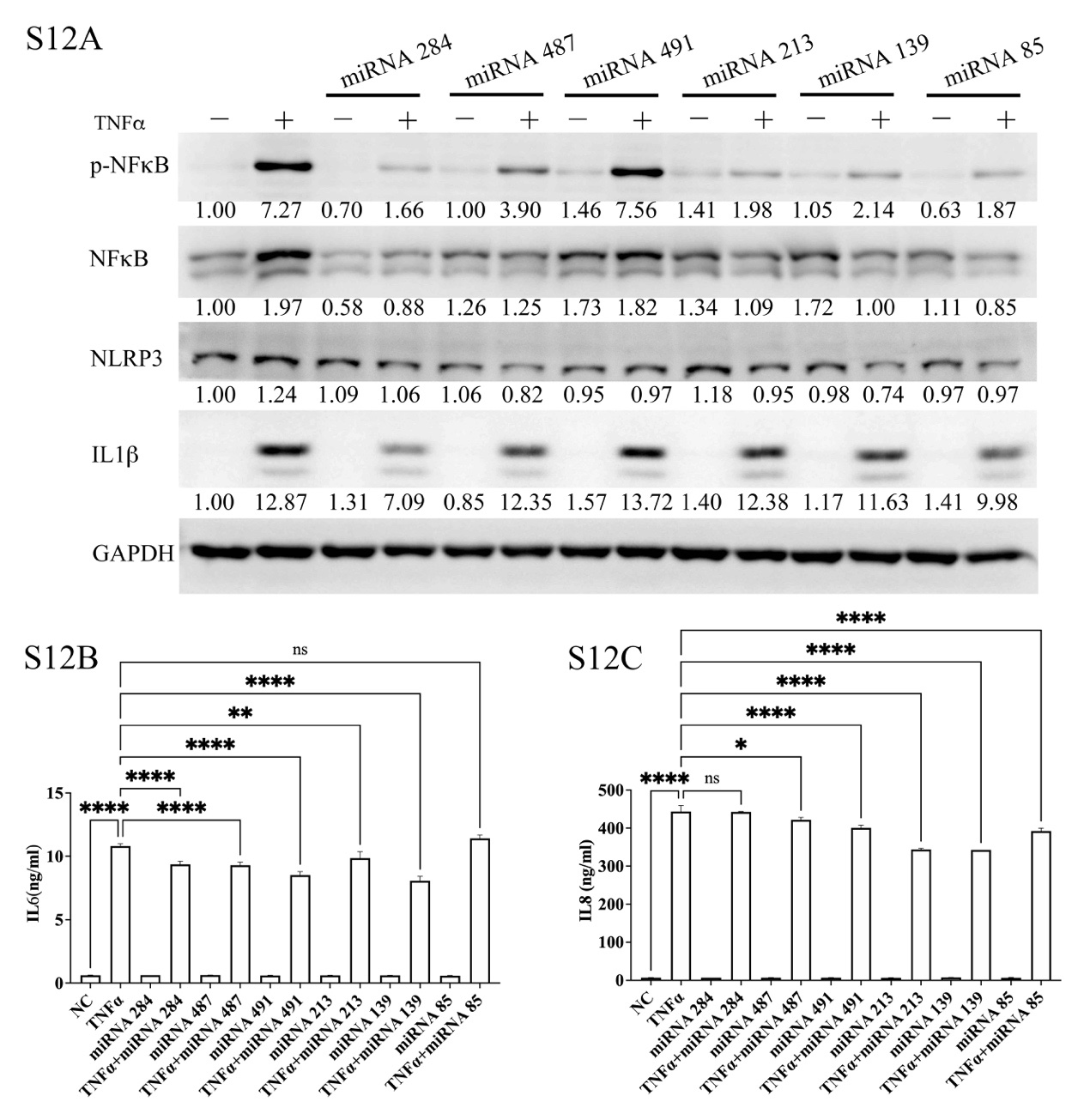


Supplementary Figure 12: *Lactiplantibacillus Plantarum* EP21 carries miRNAs that inhibit inflammation in retina pigment epithelial cells. (A) RPE-1 cells were transfected with 6 different miRNAs including miRNA 284, miRNA 487, miRNA 491, miRNA 213, miRNA 139 and miRNA 87. RPE-1 cells were treated with TNFα for 24 hr. The levels of p-NFκB, NFκB, NLRP3 and IL1β were determined by western blot. (B and C) EP21 MVs carried miRNAs that inhibited TNFα induced IL6 expression in RPE-1 cells. miRNAs were transfected into RPE-1 cells and then treated with indicated treatments for 24 hr. The IL6 (B) and IL8 (C) concentration was then determined by enzyme-linked immunosorbent assay. Analysis of variance was applied to examine for significant differences (P < 0.05), and Tukey’s multiple comparison tests were used for pairwise comparisons.


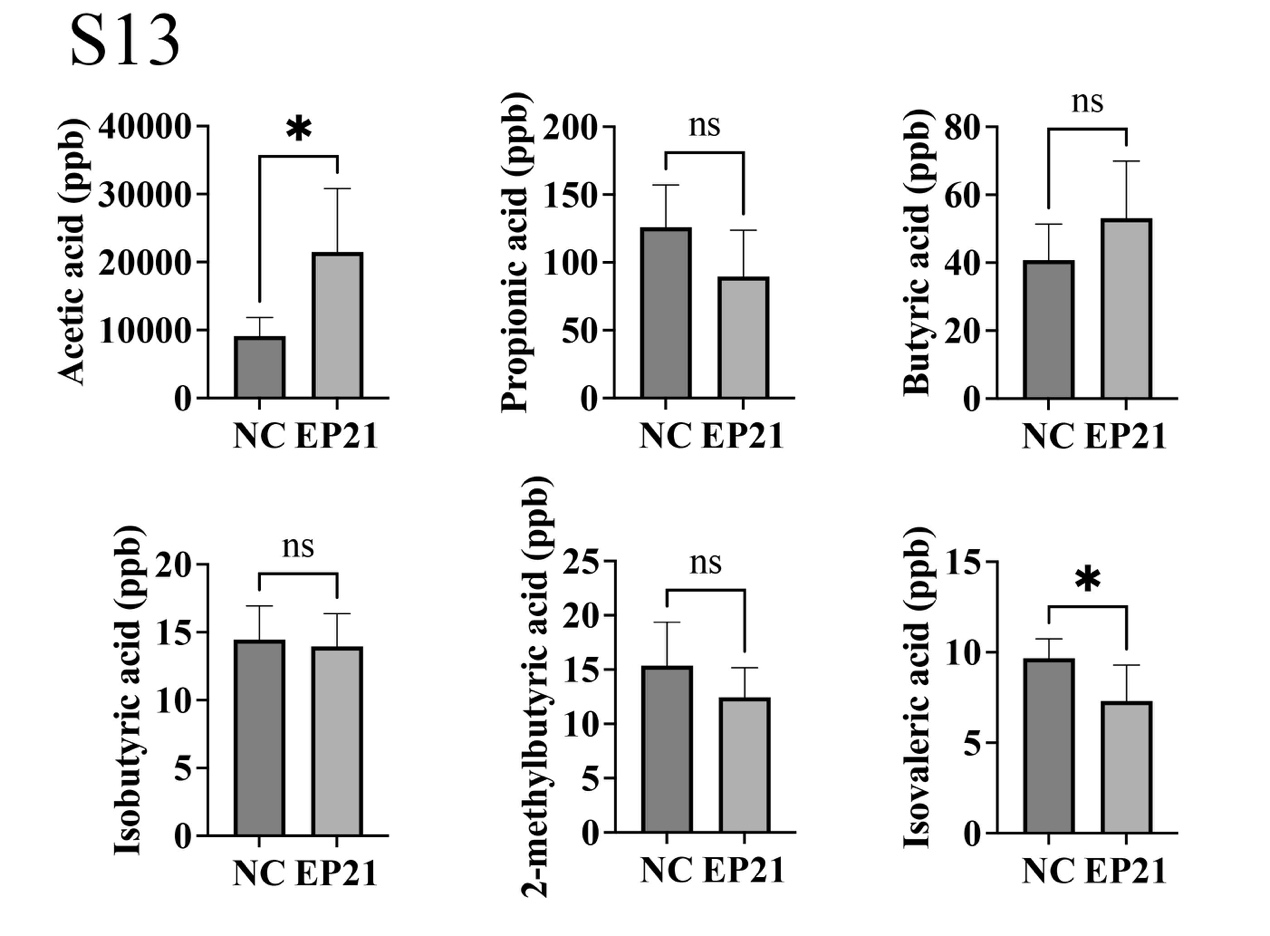


Supplementary Figure 13: *Lactiplantibacillus Plantarum* EP21 increase serum short chain fatty acids (SCFA) concentration when administered daily for 21 days in C57BL/6 mice. Sera were collected to determine the concentration of SCFA by liquid chromatograph coupled with an Agilent 1290 Infinity II UHPLC coupled with an Agilent 6495C triple quadrupole mass spectrometer (Agilent Technologies, Santa Clara, CA). T-test was used to evaluate the significant difference between negative control (NC) and EP21.


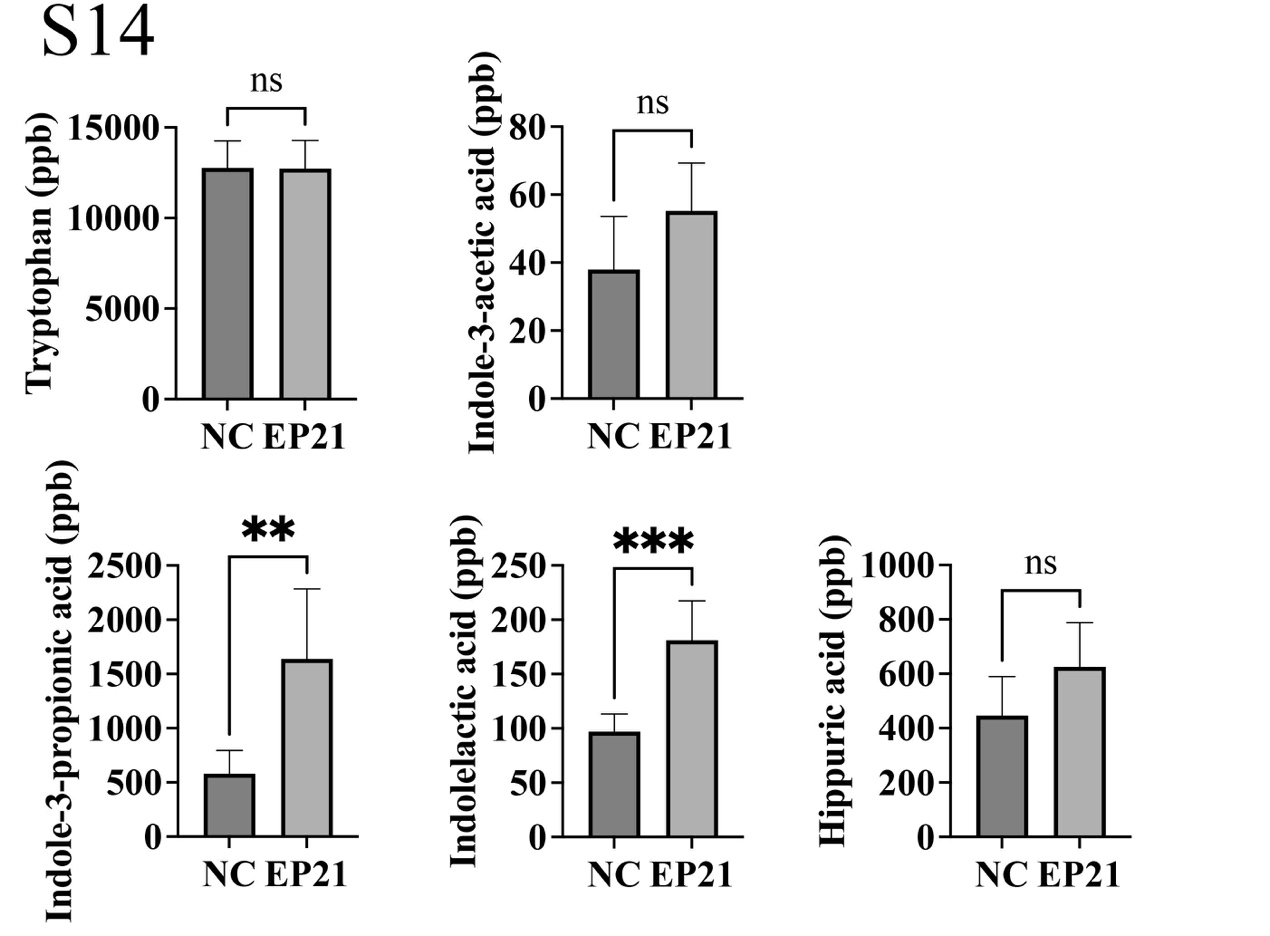


Supplementary Figure 14: *Lactiplantibacillus Plantarum* EP21 increase tryptophan metabolites concentration when administered daily for 21 days in C57BL/6 mice. Sera were collected to determine the concentration of tryptophan metabolite by liquid chromatograph coupled with an Agilent 1290 Infinity II UHPLC coupled with an Agilent 6495C triple quadrupole mass spectrometer (Agilent Technologies, Santa Clara, CA). T-test was used to evaluate the significant difference between negative control (NC) and EP21.


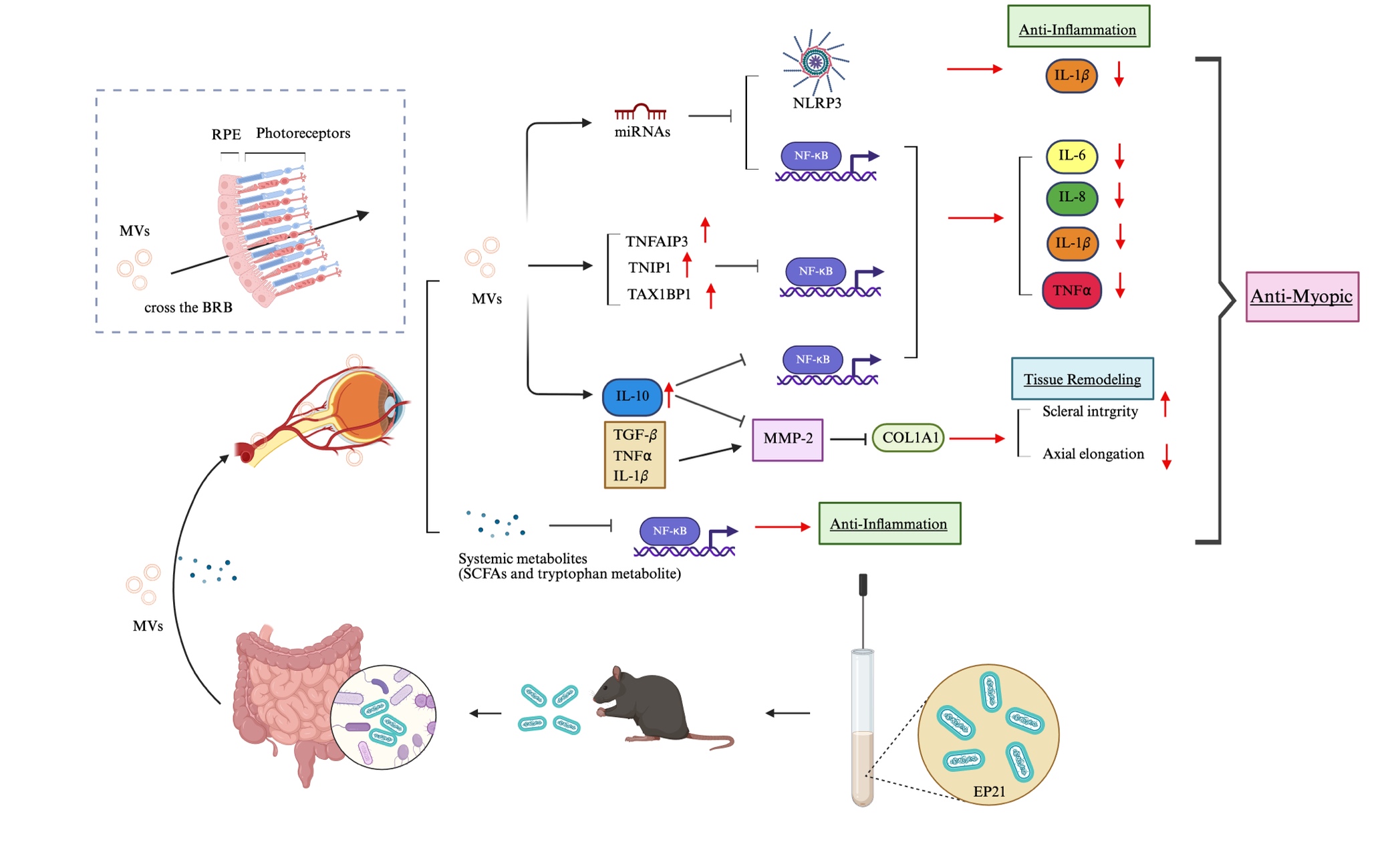


Supplementary Figure 15: A schematic representation delineates the molecular pathways through which *Lactiplantibacillus Plantarum* EP21 mediates the inhibition of myopia development
